# Realized Genetic Gain in Rice: Achievements from Breeding Programs

**DOI:** 10.1186/s12284-023-00677-6

**Published:** 2023-12-15

**Authors:** Fallou Seck, Giovanny Covarrubias-Pazaran, Tala Gueye, Jérôme Bartholomé

**Affiliations:** 1 Rice Breeding Innovation Platform, International Rice Research Institute, DAPO Box7777, Metro Manila, Philippines; 2 University Iba Der Thiam of Thiès, GrandStanding, Thiès, Senegal; 3 CIRAD, UMR AGAP, Cali, Colombia; 4 AGAP, Univ Montpellier, CIRAD, INRA, Montpellier SupAgro, Montpellier, France; 5 Alliance Bioversity-CIAT, Cali, Colombia

**Keywords:** Genetic gain, Response to selection, Breeder’s equation, Rice, Breeding program

## Abstract

Genetic improvement is crucial for ensuring food security globally. Indeed, plant breeding has contributed significantly to increasing the productivity of major crops, including rice, over the last century. Evaluating the efficiency of breeding strategies necessitates a quantification of this progress. One approach involves assessing the genetic gain achieved through breeding programs based on quantitative traits. This study aims to provide a theoretical understanding of genetic gain, summarize the major results of genetic gain studies in rice breeding, and suggest ways of improving breeding program strategies and future studies on genetic gain. To achieve this, we present the concept of genetic gain and the essential aspects of its estimation. We also provide an extensive literature review of genetic gain studies in rice (*Oryza sativa* L.) breeding programs to understand the advances made to date. We reviewed 29 studies conducted between 1999 and 2023, covering different regions, traits, periods, and estimation methods. The genetic gain for grain yield, in particular, showed significant variation, ranging from 1.5 to 167.6 kg/ha/year, with a mean value of 36.3 kg/ha/year. This translated into a rate of genetic gain for grain yield ranging from 0.1% to over 3.0%. The impact of multi-trait selection on grain yield was clarified by studies that reported genetic gains for other traits, such as plant height, days to flowering, and grain quality. These findings reveal that while breeding programs have achieved significant gains, further improvements are necessary to meet the growing demand for rice. We also highlight the limitations of these studies, which hinder accurate estimations of genetic gain. In conclusion, we offer suggestions for improving the estimation of genetic gain based on quantitative genetic principles and computer simulations to optimize rice breeding strategies.

**Supplementary Information:**

The online version contains supplementary material available at 10.1186/s12284-023-00677-6.

## Introduction

Plant breeding is the science of improving the genetics of cultivated plants to develop new varieties with desired combinations of traits to meet the needs of users. Breeding programs make use of crosses, evaluations, and strategies of selection in pools of germplasm with interesting attributes to achieve this goal (Acquaah 2009; Allard 1999). However, not only do breeding programs have agronomic (biotic and abiotic stress resistance, fertilization, and pest management), biological (genetics, physiology), economic (target markets, user needs), and statistical components (Luckett and Halloran 2017), but they also have increased in complexity over time, with the discovery and integration of molecular tools increasing the precision and efficiency (marker-assisted selection, QTL introgression, and, more recently, genomic selection) of the selection process (Cobb et al. 2018; Crossa et al. 2017; Siddiq and Vemireddy 2021). This complex process involves the investment of considerable resources, and it often takes a long time to develop and deliver the final product (about 8 to 10 years for an elite line in conventional programs) in the form of a released variety (Gallais 2011). However, resources and budgets are often limited in public plant breeding programs, particularly in low- and middle-income countries. It is, therefore, important to monitor the efficiency and sustainability of the program implemented to achieve the targeted objectives.

The efficiency of a plant breeding program can be evaluated based on several indicators that may be applied in one or more subcomponents of the breeding program: design (market segmentation, product profiling), engineering (population improvement and product development), and delivery (product commercialization, variety renewal) (Cobb et al. 2019; Covarrubias-Pazaran 2020). Ceccarelli, (2015) described three main methods for measuring the efficiency of plant breeding programs, one for each component of the breeding program. Efficiency may be evaluated: (i) by calculating the ratio of the number of varieties adopted by farmers to the number of crosses made at the beginning of a given breeding cycle; (ii) by calculating the ratio of the benefits generated by a new variety to the cost associated with its development; or (iii) by assessing the response to selection over a given period. The efficiency of a plant breeding program for developing high-yielding cultivars, leading to the release of at least one variety and its adoption by farmers, can be improved by crossing stringently selected parents (Huehn 2005; Witcombe et al. 2013). This indicator based on the adoption of varieties is widely used in the evaluation of plant breeding efficiency in the public sector, but it remains an inaccurate indicator of performance because only the benefits generated by variety adoption are captured, whereas non-adopted varieties may play an important role as future parents (Ceccarelli 2015). Unlike the first two indicators, the assessment of response to selection (also called genetic gain) is not widely used as a performance indicator in breeding programs or by stakeholders. Genetic gain nevertheless remains a robust indicator, including all three main components of a breeding cycle: crossing – evaluation – selection (Ceccarelli 2015; Cobb et al. 2019; Dudley 1997; Huehn 2005). In addition, other sub-indicators of performance, such as the maintenance of a suitable level of genetic diversity during breeding cycles, parent recycling time, crossing strategy, and selection method, are also incorporated into the formulation of genetic gain. However, several traits of interest (e.g., yield, earliness, resistance to abiotic stresses, milling quality) in breeding programs are quantitative traits. Assuming that such traits are under the control of a large number of genes, each with a small effect associated with a large environmental influence (Baker 1984; Falconer 1981), significant genetic gain can be achieved only after several cycles of recurrent selection (Rutkoski 2019a). Increases in production in farmers’ fields can be attributed to a combination of genetically improved cultivars (genetic component) and optimized crop management systems (agronomic or non-genetic component). A good performance indicator should, therefore, quantify the contribution of each of these components. Several studies aiming to quantify genetic and non-genetic contributions to crop production improvement for different cereals have reported that a large proportion of the increase in productivity was due to genetic efforts (Kumar et al. 2021; Laidig et al. 2014; Mackay et al. 2011; Piepho et al. 2014).

Efforts to improve yield and other agronomic traits of interest have been crucial since the Green Revolution. For rice (*Oryza sativa* L.), the start of modern breeding is generally traced back to the development of IR8 (Peng and Khushg 2003), a variety that helped address some of the challenges associated with food security. Rice is one of the most cultivated cereals worldwide, and it constitutes the staple food for more than half of the human population. Rice cultivation is a strategic element for food security and social stability, particularly in low and middle-income countries (GRiSP 2013). In 2021, global rice production was estimated at more than 787 million tons of paddy rice (FAO 2022), with about 90% of total production in Asia. Indeed, the genetic improvement in rice breeding has played a key role in achieving this level of production over the last five decades through the development and release of more productive varieties (Khush 2008; Mackill 2018; Mackill and Khush 2018; Xie and Zhang 2018). These varieties, adapted to different growing conditions, have enabled farmers to achieve high levels of production. However, despite this breakthrough, rice production faces many challenges due to decreases in the amount of arable land available for rice cultivation (Chauhan et al. 2017; Nguyen and Ferrero 2006) due to urbanization, soil erosion, salinity and acidity, and the impact of climate change, due to heat stress, drought, flooding, and water scarcity (Kim et al. 2013; Oort and Zwart 2018; Y. Xu et al. 2021). Moreover, a stagnation of grain yields has been observed lately in several rice-growing countries (Fischer and Edmeades 2010; Ray et al. 2012), and the rates of genetic gain in grain yield by rice breeding programs are considered too low to meet the increasing demand of rice. We need to increase rice production by at least 50% to feed the growing population (Ray et al. 2013). These challenges in rice cultivation highlight the need to increase the rate of genetic gain for important traits by developing high-yielding genotypes and designing efficient breeding strategies to continue the increase in grain yield gain (Cobb et al. 2019; Dingkuhn et al. 2015; Siddiq and Vemireddy 2021).

Our objectives here are: i) to provide the reader with a theoretical understanding of the genetic gain concept, facilitating identification of the principal factors affecting its estimation in the context of a breeding program; ii) to summarize the major results and conclusions of genetic gain studies in rice breeding over the last six decades, together with the limitations of these studies; and iii) to suggest ways of improving the estimation of genetic gain in breeding programs and for future studies. The first part of this study focuses on the principles and theoretical foundations of genetic gain and presents methods for its estimation in breeding programs. The second part provides an in-depth review of studies on the realized genetic gain in rice, mostly for grain yield and other traits of economic importance. Finally, we describe ways to improve genetic gain with tools to optimize breeding strategies.

## Genetic Gain

Genetic gain is a concept that may appear simple. However, its estimation within a breeding program remains complex, and its implementation can be complicated. It is, therefore, important to understand its theoretical basis to improve evaluations of its implications for crop breeding programs. In this section, we provide readers with a definition of genetic gain and a description of the principal methods used for its estimation.

### Definition of Genetic Gain

**Fig. 1:**
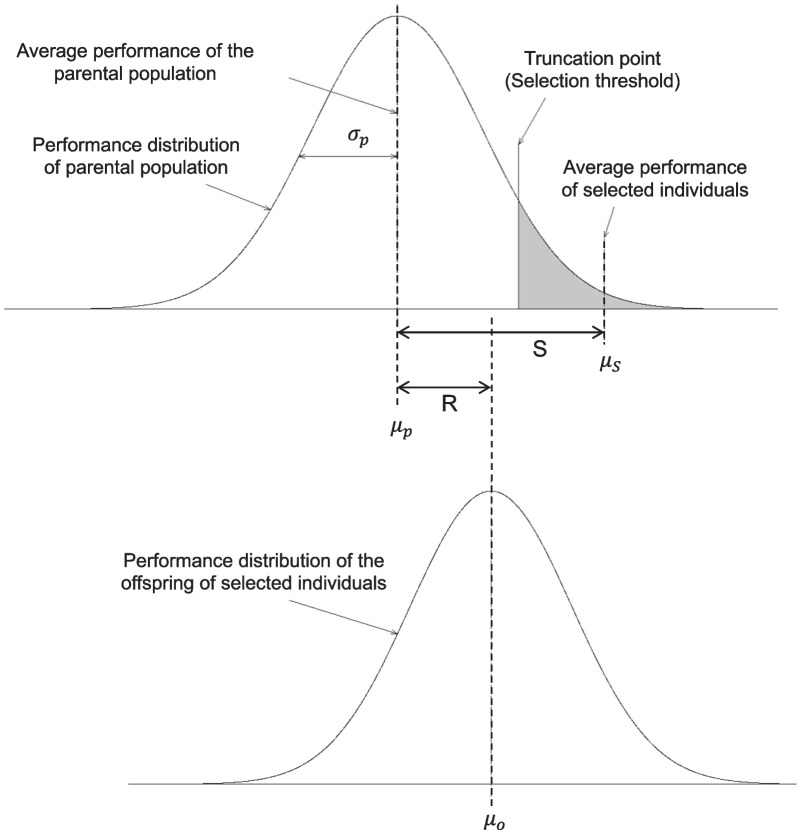
Distributions of phenotypic values of the base population, the selected individuals, and their offspring. μ_p_: mean of the parental generation, μ_s_: mean of selected individuals, S: selection differential, μ_o_ mean of the offspring generation, R: genetic gain

Selection leads to many changes in the genetic properties of a population, the most important for the breeder being the change in the average performance of the population. This change is referred to as the response to selection, denoted as $R$. The response to selection, or the genetic gain from selection, is the difference between the average performance (phenotypic, estimated breeding value or index) of the progeny of selected individuals (${\mu }_{o}$) and that of the initial population (${\mu }_{p}$) before selection (Falconer 1981): $R= {\mu }_{o}-{\mu }_{p}$. In other words, it can be described as the expected or realized intergenerational change in the average phenotypic value or genetic value of a population over at least one cycle of selection for a single trait or multiple economically important traits combined into an index in a relatively closed population (Rutkoski 2019a). Genetic gain is achieved by using only the best individuals with performances above a specific threshold for breeding. For the prediction of the genetic gain achieved over one or several breeding cycles, a relationship is established between the performances of the selected parents and their offspring (Fig. 1). The average performance of offspring can be predicted from that of their parents by linear regression with the following equation: ${\mu }_{o}={\beta }_{OP}{ \mu }_{p}$ where ${\beta }_{OP}$ is the parent–offspring regression coefficient. In the quantitative genetics model, ${\beta }_{OP}$ is equal to the narrow-sense heritability $({h}^{2}$) of the trait if selection is applied to both parents or half the heritability ${(\frac{1}{2}h}^{2})$ if selection is applied only to one of the parents. The difference between the average performance of the selected parents (${\mu }_{s}$) and the average for the whole population (${\mu }_{p}$) is known as the selection differential, denoted $S={\mu }_{s}-{\mu }_{p}$. If we center R and S on ${\mu }_{p}$ they become the mean deviation of the offspring from the population mean and the mean phenotypic value of the selected parents, respectively, both expressed as a deviation from the population mean. Finally, the equation for the response to selection, commonly known as the breeder's equation, becomes: $R= {h}^{2}S$ where ${h}^{2}$ is the heritability of the target traits and $S$ is the selection differential. Given the relationships between the components of a breeding scheme, other formulas can be derived from the classical breeder's equation. $S$ can be expressed as a function of the intensity of selection ($i$), which depends on the percentage of individuals selected and is defined as $i=\frac{S}{{\sigma }_{P}}$ where ${\sigma }_{P}$ is the square root of the phenotypic variance. Thus, $R= i h{ \sigma }_{a}$. Genetic gain may also be expressed per unit time, in which case it is described as the rate of genetic gain ($\Delta G$), the most widely used equation for expressing genetic gains: $\Delta G=\frac{i h {\sigma }_{a}}{L}$, where $L$ is the number of years required to complete one breeding cycle. The main direct application of these equations is in predicting the response to selection (Baker 1984; Falconer 1981).

### Expected and Realized Genetic Gain

Expected genetic gain is defined as the predicted change in mean phenotypic value that would be caused by a change in the genetic value of the population under a given breeding strategy (Rutkoski 2019a). Basically, it is an a priori estimate of the genetic gain from a breeding scheme. It can be estimated from the breeder's equation provided that parameters such as heritability, genetic variance, and selection intensity are known (Covarrubias-Pazaran 2020; Falconer 1981). However, under the real conditions of a large-scale breeding program, it is difficult to meet this assumption to obtain an accurate estimate of the expected gain (it is assumed that the parameters of the equation remain constant over cycles). In reality, selection intensity varies over cycles, and genetic variance tends to decrease over time (Bouffier et al. 2008; Briggs and Goldman 2006; Bulmer 1971, 1976; Dudley 2007). It is, therefore, difficult to estimate the expected gain over multiple breeding cycles accurately. Nevertheless, several plant breeding programs use the expected gain as a metric for comparing different breeding strategies (Abidine Fellahi et al. 2020; Heffner et al. 2010; Helms and Hammond 2006). Analyses can be conducted with deterministic simulation models to guide choices in the design of breeding strategies, such as the number of crosses, cycle duration, or the intensity of selection at different stages (Atlin and Econopouly 2021).

**Fig. 2:**
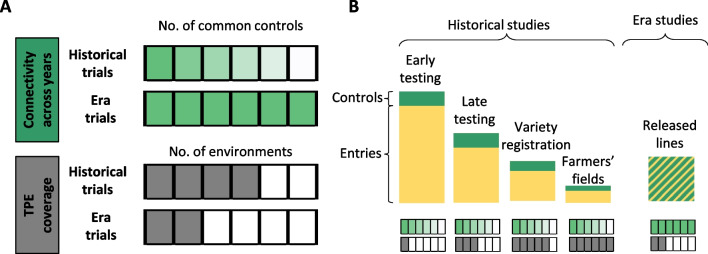
Graphical representation of the level of connectivity between entries and target population of environments (TPE) coverage associated with each type of data and stage of evaluation. A: Connectivity levels of historical studies and era studies driven by control strategy. For connectivity between years, the intensity of the green color reflects the number of common controls between trials, the higher the intensity, the greater the connectivity. For TPE coverage, the gray boxes represent the proportion of environments (year and/or location) covered by the trials conducted in each type of study. B: Genetic material used in each stage of evaluation for historical studies and era studies, with their level of connectivity and TPE coverage

Realized genetic gain is defined as the change in average population performance observed over at least one cycle of selection. Realized genetic gain can be estimated by a linear regression analysis of the average performance of populations from each selection cycle over the total number of cycles or years (Eberhart 1964; Rutkoski 2019a). The linear regression coefficient thus represents the rate of realized genetic gain per breeding cycle or year. When measuring realized genetic gain, it is commonly presented in two ways: absolute gain, which is measured in phenotypic units per cycle or year, and relative gain, which is expressed as a percentage compared to a baseline. In plant breeding, several methods for estimating realized genetic gain, with different response variables for the regression, have been described. Realized gain over a given period is estimated with experimental data of two main types: era studies or historical studies (Covarrubias-Pazaran 2020). In era studies, released varieties (or advanced lines) representing the breeding effort over a period of time are evaluated in the same environments in specific experiments. These varieties, released over the years, are assumed to represent the improvements in germplasm in each breeding cycle. For historical studies, data generated over the years by the breeding program or by the variety release system are compiled. Such data may be derived from various sources (early or advanced trials, variety registration, or on-farm trials), leading to the use of different analytical approaches (Laidig et al. 2014; Mackay et al. 2011; Rutkoski 2019a). The use of historical phenotypic data or phenotypic data from era trials can provide accurate estimates of the true rate of realized genetic gain, provided that two important characteristics of the datasets are carefully considered: connectivity between experiments/trials and TPE (target population of environments) coverage (Covarrubias-Pazaran 2020; Rutkoski 2019b). Connectivity is defined as the degree of overlap between different cohorts in the same year; it can be used to dissociate the environmental effect (year or location) from the genetic effect. Data connectivity differs between data sources. The connectivity of data from era studies is good, by definition, as the whole panel is evaluated simultaneously (Fig. 2A). By contrast, connectivity is generally much lower for historical data, mostly due to the lack of connectivity between the germplasm pools evaluated in each cohort. However, connectivity in such data can be improved by adopting a good check strategy on trials or incorporating a relationship matrix (based on pedigree or markers) into the analysis. The TPE is the target set of environments; it can be used to capture genotype x environment (GxE) interactions. TPE coverage is generally low in era studies because trials are often performed at a single site (often an experimental station). The TPE coverage of historical data depends on the type of trial. It is low for early trials, moderate for advanced trials, and high for variety registration and on-farm trials (Table 1, Fig. 2B).

**Table 1:** Type of genetic gains that can be estimated by a breeding program, sources of datasets and the characteristics of each type of population used in terms of connectivity and TPE coverage. Modified from Covarrubias-Pazaran (2020)

Method	Data required	Type of material	Factors to be considered	Connectivity^a^/TPE^b^ coverage
Expected	Any trial information	Any generation material	The heritability used will have an important effect on the under- or overestimation of the metric	Low after first selection cycle
Realized	Era trial information	Early material	1) TPE coverage is low (usually a few locations & a couple of years). 2) Connectivity between entries is maximal (cohorts evaluated at the same time). 3) Sample can overestimate the metric	High/Low
		Advanced material		
		Released varieties		
		On-farm		
	Historical trial information	Early material	1) TPE coverage can be low (early), intermediate (advanced) or high (varieties). 2) Connectivity between entries depends on controls and the use of methods such as EBV. 3) Sample can overestimate the metric	Variable/Low
		Advanced material		Variable/intermediate
		Released varieties		Variable/High
		On-farm		Variable/High

^a^Connectivity: The degree of overlap of different cohorts in the same year

^b^TPE: Target population of environments. The better the coverage of TPE, the more accurate the estimates of genetic and breeding value we can obtain

### Statistical Models

Assuming that the genetic trend resulting from selection is linear during consecutive early cycles of selection (Eberhart 1964; Hallauer et al. 2010; Rutkoski 2019a), the realized genetic gain for a quantitative heritable trait can be estimated with the following regression model:

$${Y}_{i}= {\mu }_{0}+\beta {x}_{i}+{\varepsilon }_{i}$$

where ${Y}_{i}$ is the observed population performance for a selection cycle; ${\mu }_{0}$ is the estimated average performance of the initial population; $\beta$ is the linear regression coefficient representing the rate of genetic gain per unit of phenotypic value per cycle (or per year); ${x}_{i}$ corresponds to the selection cycle and ${\varepsilon }_{i}$ to deviations from the regression model. If a large number of breeding cycles are performed, and gene frequencies are very low in the initial population, a non-linear trend can be expected, and, in such cases, the simple linear model described above should be extended by including quadratic and cubic terms (Eberhart 1964). More accurate estimates of the model parameters (${\mu }_{0}; \beta ; {\varepsilon }_{i}$) can be obtained by including in the model other factors (fixed and random effects), as a function of the data and experimental design used (Table 2).

**Table 2:** Summary of linear mixed models and linear regression models frequently used for the estimation of realized rate of genetic gain in plant breeding programs

Method	Model	Effects	References
Era trial	${y}_{i}=\mu +\beta {r}_{i}+{\varepsilon }_{i}$	Phenotypic value (y) Regression coefficient (β) Year of release (r)	Duvick (2005), Hallauer et al. (2010), Rutkoski (2019b)
EBV^b^	${y}_{ijk}=\mu +{g}_{i}+{d}_{i}+{l}_{k}+{\varepsilon }_{ijk}$ ${g}_{i}=\mu +{\beta r}_{i}+{\varepsilon }_{i}$	Phenotypic value (y) Genotype effect (g)^a^ Location (L) Year (d) Regression coefficient (β) Breeding cycle (r)	Garrick (2010), Rutkoski (2019b)
Variety registration trial	${y}_{ijk}=\mu +{g}_{i}+{\beta r}_{i}+{d}_{j}+{\gamma t}_{i}+{l}_{k}+{h}_{jk}+{x}_{ij}+{z}_{ik}+{\varepsilon }_{ijk}$	Phenotypic value (y) Genotype (g)^a^ Location (l)^a^ Year (d)^a^ Regression coefficients for genetic and non-genetic trends ($\beta et \gamma$) G x Location (z)^a^ G x Year (x)^a^ Location x Year (h)^a^	Laidig et al. (2014), Piepho et al. (2014), Rutkoski (2019b)

^a^Random variables that are independent and identically distributed (iid), assumed to be normal

^b^Estimated breeding value. In the EBV method, the first model is used to estimate breeding value and the second model is used to estimate genetic gain (simple linear regression coefficient)

**Fig. 3:**
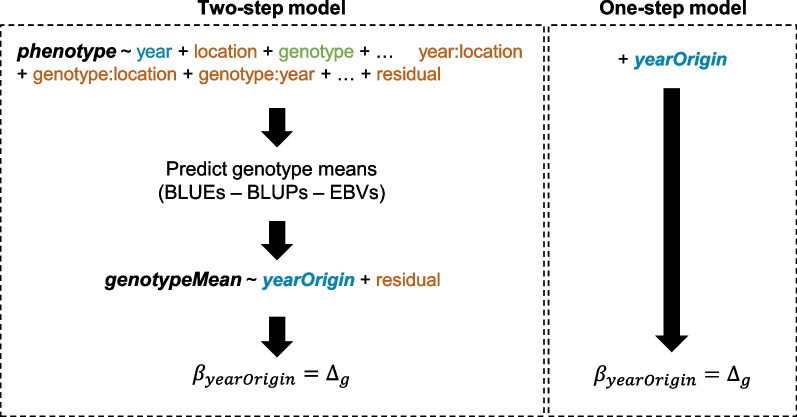
Two methods for estimating realized genetic gain using regression models: one-step and two-step. In the model, the numerical variables are in bold, and the factors are in normal font. The different class colors define the class of factors. Blue represents variables set as fixed effects in the model, red represents variables set as random effects, and green represents variables that may be set as random or fixed effects depending on whether one is expecting to estimate the BLUEs or BLUPs

The average phenotype collected directly based on the evaluated genotypes, or genetic means estimated from these phenotypes can be used as the response variable in the regression model for the genetic trend over time. The regression slope corresponding to the realized genetic gain can be estimated in different ways. In general, a concomitant estimation of the slope is performed in a single model with a fixed regression term, in a single-step process (Fig. 3). The regression coefficient can also be estimated separately by fitting several mixed models before the regression model. This two-step approach makes it possible to estimate the adjusted genetic means, which can then be used as the response variable in the regression model for the estimation of the rate of genetic gain (Fig. 3). The adjusted means may be estimated from the following linear mixed model:

$${Y}_{ijk}=\mu +{g}_{i}+{y}_{j}+{l}_{k}+{y:l}_{jk}+{g:l}_{ik}+{g:y}_{ij}+{\varepsilon }_{ijk}$$

where ${Y}_{ijk}$ is the observed phenotypic value of genotype $i$ in year $j$ and at location $k$; ${g}_{i}$ is the effect of the *i*th genotype; ${y}_{j}$ is the random effect of the *j*th year; ${l}_{k}$ is a random effect of the *k*th location; ${y:l}_{jk}$ is the random effect of the interaction between year and location; ${g:y}_{ij}$ is a random effect of the interaction between genotype and year; ${g:l}_{ik}$ is a random effect of the interaction between genotype and location, and ${\varepsilon }_{ijk}$ is the random residual error of the model. Genotype effects may be considered to be fixed for fitting a best linear unbiased estimator (BLUE) or random for BLUP (best linear unbiased predictor). A relationship matrix can be incorporated with the random genotype effects to obtain estimated breeding values (EBVs).

Furthermore, as the observed trend is a result of both genetic effects due to breeding efforts and non-genetic effects due to the improvement of agricultural practices, models have been developed to separate these two components. Piepho et al. (2014) proposed a two-step method for estimating the genetic gain captured by the first year of testing for lines and the non-genetic gain captured by the calendar year:

$${Y}_{ijk}=\mu +{G}_{i}+{L}_{j}+{A}_{k}+{(LA)}_{jk}+{(GL)}_{ij}+{(GA)}_{ik}+{\left(GLA\right)}_{ijk}+{\varepsilon }_{ijk}$$

where ${Y}_{ijk}$ is the mean yield of the $i$ th genotype at the $j$ th location in the $k$ th year, $\mu$ is the overall mean, ${G}_{i}$ is the main effect of the $i$ th genotype, ${L}_{j}$ is the main effect of the $j$ th location, ${A}_{k}$ is the main effect of the $k$ th year, ${(LA)}_{jk}$ is the $j$ th location × *k*th year interaction effect, ${(GL)}_{ij}$ is the $i$ th genotype × *j*th location interaction effect, ${(GY)}_{ik}$ is the $i$ th genotype × *k*th year interaction effect, ${\left(GLA\right)}_{ijk}$ is the interaction of the $i$ th genotype with the $j$ th location in the $k$ th year and ${\varepsilon }_{ijk}$ is the residual term. With the exception of $\mu$, ${G}_{i}$ and ${A}_{k,}$ all effects are assumed to be random and independently distributed, with a constant variance. The regression term for the rate of genetic gain is as follows:

$${G}_{i}=\beta {r}_{i}+{H}_{i}$$

where $\beta$ is a fixed regression coefficient for the genetic trend; ${r}_{i}$ is the first testing of genotypes and ${H}_{i}$ is the random deviation from the genetic trend line, with ${H}_{i} \sim (0, {\sigma }_{H}^{2})$. The non-genetic trend is:

$${A}_{k}=\gamma {t}_{k}+{Z}_{k}$$

where $\gamma$ is a fixed regression coefficient for the non-genetic trend, ${t}_{k}$ is the continuous covariate for the calendar year and ${Z}_{k}$ is the random residuals for the agronomic trend with ${Z}_{k} \sim (0, {\sigma }_{Z}^{2})$.

### Uses of the Term Genetic Gain

The terms "genetic gain" or "response to selection" are often used inaccurately to describe the genetic part of a trait's evolution over time, whatever the source of the material. However, this trend is not always the result of selection cycles of a specific breeding program within a relatively closed population (Rutkoski 2019a). Some studies use a very broad definition of genetic gain, sometimes referring to the evolution over time of the phenotypic characteristics of advanced lines or widely cultivated varieties resulting from different breeding programs. In this case, the trend observed does not reflect the performance of a breeding program but rather the contribution of genetic improvement to the progress made, whether in yield or another trait. Therefore, analyzing historical data from national or regional official variety trials (de la Vega et al. 2007; Feng et al. 2017; Laidig et al. 2014; Mackay et al. 2011; Muralidharan et al. 1996, 2002, 2019, 2022; L. Xu et al. 2020) or comparing popular varieties from several institutes from different periods (Liu et al. 2021; Meng et al. 2021, 2022; Xiao et al. 2012; Yadav et al. 2021; Zeleke et al. 2021; Zhu et al. 2016) does not necessarily reflect the genetic gain achieved by a breeding program or provide any indication of the performance a breeding program. A more accurate term to describe the impact of selection on a given trait at different levels, which may involve materials from various breeding programs, is genetic improvement or genetic progress. In this study, we focused on genetic gain as an indicator of breeding program performance. Studies on genetic progress are mentioned for comparison purposes.

## Realized Genetic Gain for Major Traits in Rice

Increasing numbers of studies in recent years have estimated the genetic gain from rice breeding programs. The key challenges facing breeding programs are evaluating the efficiency of the strategy used to optimize resource allocation, improving the rate of genetic gain, and developing high-yielding varieties. Most studies on genetic gain in rice have focused on grain yield. However, other traits have been studied, albeit to a lesser extent: plant height, days to flowering, grain quality, yield components, disease resistance, and physiological traits. In this section, we provide a detailed overview of the literature and present the genetic gain achieved by rice breeding programs over the last six decades.

### General Overview

**Fig. 4:**
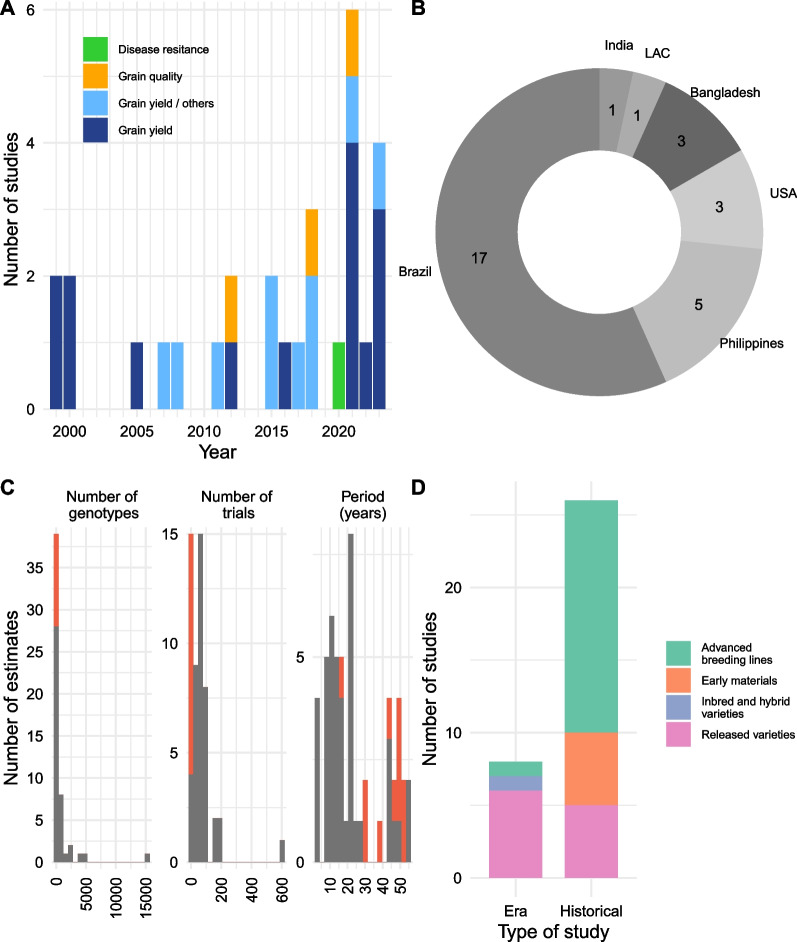
Summary of the literature on genetic gain in rice. Panel (**A**) shows the number of studies published each year. Panel (**B**) indicates the distribution by country of the studies (LAC: Latin America and the Caribbean). Panel (**C**) details the number of genotypes, the number of trials, and the period used for the various estimates of the rate of genetic gain. In grey are the historical studies, and in red are the era studies. Panel (**D**) indicates the distribution of types of material between era and historical studies. More information on the studies summarized in this figure is available in Tables S1 and S2

We reviewed 29 studies on genetic gains in rice, published between 1999 and 2023, with a large number of the studies published recently (Fig. 4A). These studies covered a wide range of traits of interest. Grain yield was, inevitably, the most studied trait: 15 of the 29 studies focused on grain yield only, ten assessed grain yield in combination with other traits (mainly plant height and days to flowering), and four studies analyzed traits other than grain yield, mostly related to grain quality (Fig. 4A, Table 3). The studies covered diverse ecosystems, including irrigated, rainfed upland and lowland, drought-prone, and salinity-prone environments, with a majority focused on irrigated ecosystems (Table 3). Most studies (27) considered a single ecosystem, but some compared the realized genetic gains between different ecosystems (Khanna et al. 2022; Kumar et al. 2021). The studies considered also covered different rice-growing regions worldwide, with considerable variability in the number of studies carried out per country or region (Fig. 4B). Brazil was the country with the largest number of studies (17), followed by the Philippines (5), then the United States (3), and Bangladesh (3). Fewer than three studies represented other countries or regions. The studies used different data types: historical data from a breeding program for 22 and era data for seven studies. None of the studies used both data types. The numbers of genotypes and trials also varied considerably between studies (Fig. 4C). Indeed, the number of genotypes evaluated ranged from six (Peng et al. 2000) to 15,286 (Juma et al. 2021), with a sharp contrast between era and historical studies. Most era studies were based on fewer than 50 genotypes. Only one era study included substantially more genotypes, 284 in total (Cruz et al. 2021). Conversely, historical studies used larger sets of genotypes to estimate the rate of genetic gain. The number of trials was also greater on average in studies based on historical data. For era studies, the number of trials ranged from one (Peng et al. 2000) to four (Cruz et al. 2021; Souza et al. 2007; Streck et al. 2018a; Venkatanagappa et al. 2021), whereas for historical studies, it ranged from five (Pinson et al. 2012) to 603 (Breseghello et al. 2011). As expected, the material evaluated was also more diverse in historical studies, including early material, advanced lines, and released varieties (Fig. 4D). The periods assessed in these studies also varied widely, ranging from 3 to 55 years, with the shortest periods generally corresponding to historical studies (19 years on average) and the longest periods corresponding to era studies (40 years on average, Fig. 4C). More detailed information about the 29 studies, including the estimates of genetic gain, is provided in Additional file 1: Tables S1 and S2.

**Table 3:** Set of studies on genetic gain in rice described in this article

Study type	Ecosystem	Traits	No. of studies^a^	References
Historical	Irrigated	Grain yield	10	Biswas et al. (2023), Breseghello et al. (1999), DoVale et al. (2012), Juma et al. (2021), Khanna et al. (2022), Kumar et al. (2021), Rahman et al. (2023), Rangel et al. (2000), Silva Júnior et al. (2021), Soares et al. (2005)
		Grain yield, days to flowering, plant height	4	da Costa et al. (2021), dos Reis et al. (2015), Morais Júnior et al. (2017), Streck et al. (2018b)
		Grain quality	1	Pinson et al. (2012)
	Upland	Grain yield, days to flowering, plant height	4	Barros et al. (2018), Breseghello et al. (2011), Morais Júnior et al. (2015), Pereira de Castro et al. (2023)
		Disease resistance	1	Alves et al. (2020)
		Grain yield	1	Soares et al. (1999)
	Drought prone	Grain yield	2	Khanna et al. (2022), Kumar et al. (2021)
	Salinity prone	Grain yield	1	Khanna et al. (2023)
Era	Irrigated	Grain yield	3	Peng et al. (2000), Samonte et al. (2016), Venkatanagappa et al. (2021)
		Grain quality	2	Cruz et al. (2021), Streck et al. (2018a)
		Grain yield, days to flowering, plant height	1	Tabien et al. (2008)
	Upland	Grain yield, days to flowering, plant height	1	Souza et al. (2007)

^a^Some studies reported estimates of genetic gain for several ecosystems

Concentrating exclusively on studies which focus on grain yield (Table 3), 25 studies reported genetic gain for grain yield: five studies were based on era data (Peng et al. 2000; Samonte et al. 2016; Souza et al. 2007; Tabien et al. 2008; Venkatanagappa et al. 2021), and 20 were based on historical data. For the era studies, population size ranged from six (Peng et al. 2000) to 44 genotypes (Venkatanagappa et al. 2021). In most era studies, the set of genotypes evaluated consisted of released varieties. However, to increase the accuracy of the evaluation, breeding lines or external cultivars were sometimes included in the era panel, as control genotypes (Peng et al. 2000; Tabien et al. 2008). More than half the era studies were conducted exclusively on experiment stations with a maximum of two sites (Peng et al. 2000; Tabien et al. 2008; Venkatanagappa et al. 2021). The replication of trials over time (years or crop seasons) varied from a single trial (Peng et al. 2000) to four trial fields (Souza et al. 2007; Venkatanagappa et al. 2021).

Twenty of the 25 studies focusing on yield used historical data. Most studies (60%) focused on the irrigated ecosystem, with fewer dealing with rainfed, upland, salinity-prone, or drought-prone environments. The historical studies included populations of 62 (Breseghello et al. 1999) to 15,286 (Juma et al. 2021) genotypes. Fifteen studies used advanced lines but also included released varieties as control genotypes to increase the connectivity between the various trials. Four studies used early materials to evaluate the performance of recurrent selection and its ability to generate successful lines (Barros et al. 2018; Morais Júnior et al. 2015, 2017; Pereira de Castro et al. 2023). The historical trials were conducted mostly on-station, in standard growing conditions, and most were multi-environment trials. The time window covered by the historical studies ranged from 4 to 55 years, with 13 of the 20 studies covering periods of 10 to 20 years.

### Important Findings

#### Large Genetic Gains Can be Achieved for Grain Yield

The estimates of the genetic gain achieved in the 25 studies on grain yield were highly variable (Table 4), ranging from 1.5 kg/ha/year (Khanna et al. 2023) to a maximum of 167.6 kg/ha/year (Silva Júnior et al. 2021) over all ecosystems. This wide variation is the consequence of several interacting factors: the type of study (era vs. historical trials), the genetic material (advanced vs. early-stage material), the period studied, the statistical model, and agronomic management. This makes it difficult to identify the determinants of larger genetic gains directly from these studies. However, a few interesting points can be highlighted. First, the upper limits for these estimates demonstrate the potential of rice breeding programs to achieve large genetic gains for yield in different ecosystems. Indeed, a rate of genetic gain exceeding 1.5% was reported in studies in all the major ecosystems. Second, these studies focused on “short” periods (i.e., less than 20 years), which are more relevant for the monitoring of breeding programs as the estimates reflect breeding decisions. The authors tried to increase the accuracy of estimates of genetic gain by splitting the genotypes into maturity groups or by region of origin or breeding phase, as the numbers of datasets analyzed in historical studies were large (from 10 to more than 500). This analytical approach provides a better assessment of the program's performance as a function of its breeding objectives. Third, the four studies based on the evaluation of early material were among those in which genetic gain was greatest (Barros et al. 2018; Morais Júnior et al. 2015, 2017; Pereira de Castro et al. 2023). For example, for a recurrent selection scheme based on the recycling of S_1:2_ families (Morais Júnior et al. 2017), the mean gain over three breeding cycles was 153 kg/ha/year (1.98%). These results provide support for the notion that shorter breeding cycles contribute to a higher rate of genetic gain.

**Table 4:** Rate of realized genetic gain for grain yield for each ecosystem according to the type of study (era or historical). More detailed information on the studies summarized here is provided in supplemental file 1: Table S1

Study type	Ecosystem	No. of estimated values	Genetic gain (kg/ha/year)		
			Mean^a^	Min	Max
Historical	Irrigated	23	40.3	6.7	167.6
	Upland	7	47.3	19.1	78.8
	Salinity prone	4	5.9	1.5	14.0
	Drought prone	3	12.9	2.3	27.0
Era	Irrigated	7	43.4	17.4	81.0
	Upland	2	21.1	14.6	27.6

^a^Calculated values

#### A slowing of Genetic Gain for Yield in Recent Years?

Most of the studies concluded that yield had progressed significantly over a long period, but a closer look at the different phases of the breeding programs highlighted a mixed trend in genetic gain for grain yield. For example, a stagnation in the rate of genetic gain was observed recently for the IRRI irrigated rice program (Juma et al. 2021; Venkatanagappa et al. 2021). Peng et al. (2000) evaluated varieties originated from the program (between 1966 and 1995) and found an annual gain of 75 to 81 kg/ha/year, equivalent to an annual increase of 1%. Using an era study including more recent material from the same program (1966 – 2016), Venkatanagappa et al. (2021) estimated the annual gain at 17.35 kg/ha/year to 20.23 kg/ha/year, corresponding to an annual gain of only 0.41% to 0.55%. Using historical data from the IRRI irrigated rice program, Juma et al. (2021) observed a similar slowing of the rate of genetic gain for yield. Indeed, the estimate was 8.75 kg/ha/year (0.23%) for the period 1964–2014, with the last ten years presenting a plateau. Using historical data from India, Kumar et al. (2021) reported a decrease in the performance of advanced material during the last two years of their study in irrigated and severe stress conditions. A similar trend was observed for the irrigated rice breeding program in Minas Gerais, Brazil. According to da Costa et al. (2021), a deceleration in genetic gain for yield was observed, with values decreasing from 88.66 kg/ha/year between 1993 – 1999 to 22.69 kg/ha/year between 2010 – 2019. This corresponds to annual rates of gain of 1.62% and 0.42%, respectively. Also, in Brazil, Breseghello et al. (2021) reported a non-significant trend towards a slowing of the rate of genetic gain from 1982 to 2021. Several factors can contribute to a slowing of the rate of genetic gain for yield. Indeed, breeding programs evolve, with changes to the breeding objectives or to the population, with the introduction of new material. These factors may greatly affect the realized genetic gain. This situation is illustrated by the new plant-type approach developed at IRRI during the early 1990s (Peng et al. 1994, 2004). Productivity was lower in the first generation than in improved varieties (Peng et al. 2008), but the second generation was more successful, with better characteristics, including a higher grain yield. More generally, changes in market needs or the occurrence of major diseases or abiotic stresses can lead to changes in breeding objectives. A study by Breseghello et al. (2011) suggested that blast susceptibility played an important role in limiting the development of high-yielding genotypes due to the avoidance of crosses between high-yielding but blast-susceptible genotypes. There may have been a significant trade-off between the intensification of selection pressure for grain quality-related traits (milling, appearance, cooking, and nutritional qualities) over the last four decades and the realized genetic gain in grain yield (Barros et al. 2018; Breseghello et al. 2011; Silva Júnior et al. 2021; Streck et al. 2018b). This slowdown of the rate of genetic gain cannot be generalized to all studies. Indeed, some studies reported a steady increase in genetic gain for grain yield (Khanna et al. 2022; Pereira de Castro et al. 2023; Samonte et al. 2016; Streck et al. 2018b). This highlights the importance of long-term evaluation of genetic gain in relation to breeding strategies.

#### Other Traits Also Play an Important Role

Genetic gain for other agronomically important traits and the effects of these traits on grain yield gain are increasingly being studied to ensure continual long-term genetic gain in grain yield. Indeed, breeding decisions are not exclusively based on grain yield but also depend on several other important agronomic traits. Efforts are now being made in rice breeding programs to understand the impact of breeding decisions on these traits, particularly as relates to grain yield, through dissection of the drivers of genetic gain in grain yield, with the aim of better guiding the improvement of other traits to ensure greater gains in grain yield. Additional file 1: Table S2 summarizes the information for the studies reporting genetic gain for traits other than yield.

##### Plant Height

Yield potential is related to plant height, as shorter plants are less likely to suffer lodging and yield loss. The genetic improvement of rice has resulted in a significant decrease in plant height since the beginning of the Green Revolution. This decrease has been achieved mostly through the use of dwarfing genes (Liu et al. 2018; Peng and Khushg 2003; Siddiq and Vemireddy 2021), and has led to the design of a new plant architecture, with a transition from traditional tall varieties with moderate productivity (about 2 t ha^−1^) to highly productive semi-dwarf varieties (potential yield of 9 to 11 t ha^−1^). Most studies focusing on genetic gain in plant height reported a reduction in this trait (Barros et al. 2018; Breseghello et al. 2011; da Costa et al. 2021; Morais Júnior et al. 2015; Pereira de Castro et al. 2023; Souza et al. 2007; Streck et al. 2018b; Tabien et al. 2008). For example, Tabien et al. (2008) reported the most important decrease with up to -1.29 cm yr^−1^ for their varieties released for irrigated ecosystems between 1944 and 1992. In upland ecosystem, Souza et al. (2007) found that early-maturing material decreased in height by -0.49 cm yr^−1^ and late-maturing material by -0.71 cm yr^−1^ over 50 years. This represented a decrease of 29 cm in the early-maturing group and 42 cm in the late-maturing group. Using historical data from the upland rice breeding program in Brazil, Breseghello et al. (2011) estimated an annual decrease in height of 13 cm over 25 years (-0.52 cm yr^−1^). Similarly, Streck et al. (2018b) estimated that plant height decreased by 14 cm between 1972 and 2016 (-0.32 cm yr^−1^). For the studies based on early-generation evaluation, a reduction of plant height was also observed even after few cycles of breeding: -0.63 cm yr^−1^ (Pereira de Castro et al. 2023), -0.43 cm yr^−1^ (Morais Júnior et al. 2015), -0.11 cm yr^−1^ (Barros et al. 2018). These estimates of genetic gain for plant height are important to consider for the long-term objectives of a breeding program. Indeed, some studies have found negative correlations between plant height and grain yield (Breseghello et al. 2011; Morais Júnior et al. 2015; Pereira de Castro et al. 2023). This can lead to a limitation in genetic gain for yield. While there is no limit to the objective of increasing grain yield, rice breeding programs do not aim for a continuous reduction in plant height. Rice breeding programs are, therefore, now trying to maintain an optimum plant height, with trade-offs for productivity and lodging resistance.

##### Flowering Time

Depending on the ecosystem, agroclimatic conditions, and cropping system, farmers target an optimal maturity period. This is why days to flowering (or days to heading) has been a key breeding target for several decades. Breeding programs designed to improve materials for a wide area typically have multiple maturity groups for a given ecosystem, and several studies have reported the genetic gain for grain yield according to the groups of maturity (Soares et al. 1999; Souza et al. 2007). For more intensive irrigated or rainfed systems, different advantages have been associated with an earlier flowering date. Indeed, the selection of early-maturing cultivars has made it possible to obtain at least two growing seasons per year and to decrease the costs and exposure of crops to biotic and abiotic stresses, such as insects, pathogens, drought, and typhoons (Atlin and Econopouly 2021; Tabien et al. 2008; Vergara et al. 1966). In this context, Peng et al. (2000) estimated that the total duration of growth for cultivars released between 1974 and 1983 was ten days shorter than that for cultivars released before this period. Regarding the evaluation of genetic gain for days to flowering, several breeding programs have reported the genetic trend for this trait in order to evaluate the impact of breeding decisions. In an assessment of rice breeding in Texas, Tabien et al. (2008) found that the number of days to heading over 48 years (1944 to 1992) had decreased by 0.21 to 0.24 days per year. A similar gain was estimated in Brazil between 1984 and 2009, with a decrease of 0.25 days per year (Breseghello et al. 2011). Another study in Brazil (Streck et al. 2018b) estimated that time to heading decreased by 0.21 days per year between 1972 and 2016. More recently, it has been noticed that this trait has become stable as the breeding population's average maturity reaches the optimum (Breseghello et al. 2011; da Costa et al. 2021; Morais Júnior et al. 2015; Streck et al. 2018b).

##### Grain Quality

The improvement of rice grain quality has become an important breeding target in almost all rice breeding programs since the early 1980s. Rice grain quality has four main components: milling (*e.g.* milled rice rate, head rice recovery), appearance (*e.g.* chalkiness, grain length-to-width ratio), cooking quality (*e.g.* amylose content and gelatinization temperature), and nutritional qualities (*e.g.* protein content, zinc content) (Cruz et al. 2021; Streck et al. 2018a). The grain quality of the first high-yielding varieties developed early in the Green Revolution was poor (low head rice recovery, high percentage of chalky grain, and high amylose content), leading to efforts being made in different rice programs to improve the appearance, cooking, and eating qualities of rice varieties (Khush and Virk 2005; Mackill and Khush 2018). Despite the importance of these traits, very few breeding programs have evaluated genetic gains in grain quality. For cooking and eating quality traits, no significant increase in amylose content (0.007%) or gelatinization temperature (0.025) was observed from 1999 to 2015 in Latin America and the Caribbean (Cruz et al. 2021). However, significant genetic gain has been reported for appearance quality. In Brazil during the period 1972 – 2016, smaller decreases in the percentage of chalky grain and chalkiness area were estimated with annual gains of -0.03% and -0.14%, respectively (Streck et al. 2018a). In addition to these assessments of the genetic gain for grain quality related traits, other studies on the genetic progress have been reported on a national scale. In a study in China from nationally released varieties from 1990 to 2020, Zhou et al. (2021) reported a significant decrease in amylose content of 0.31% per year and an increase in gelatinization temperature expressed as an alkali-spreading value of 0.12 per year but no significant genetic progress has been estimated for protein content. A significant decrease of 3.15% yr^−1^ in the percentage of chalky grain, associated with a slight decrease of 0.52% yr^−1^ in the chalkiness area, has been estimated. In another example in China, Feng et al. (2017) showed contrasted results in terms of genetic progress for grain quality traits over the period 2000 – 2014. For the hybrids, significant progress was made for the degree of chalkiness, but no improvement in head rice rate was reported. The authors concluded that more efforts are needed to improve grain quality in the future.

#### Statistical Methods Have a Strong Influence

The various studies made use of a wide range of statistical approaches, mostly based on linear regression with one-step or two-step mixed model analysis (Additional file 1: Tables S1 and S2). However, only Silva Júnior et al. (2021) used two different methods, identified as the Venkosky and Breseghello methods, on the same dataset. This study analyzed data from value for cultivation and use trials conducted in Minas Gerais over a period of 23 years. The estimated gains in the three municipalities, calculated by the Venkovsky method, were 53.1 kg/ha/year, 8.68 kg/ha/year, and 6.65 kg/ha/year, corresponding to gains of 1.46%, 0.14%, and 0.11%, respectively. By contrast, Breseghello's method based on linear regression gave higher absolute gain values. Gains were estimated at 167.62 kg/ha/year, 57.88 kg/ha/year and 93.93 kg/ha/year, corresponding to 0.23%, 0.04% and 0.10%, respectively. This large difference between these two methods of estimation highlights the important contribution of the method to the variability of genetic gain. In a simulation study, Rutkoski, (2019b) evaluated five methods for estimating realized genetic gain based on their precision, efficiency, correlation between true annual mean breeding values and predicted annual mean breeding values, and absolute difference between the true and estimated realized genetic gain (error). Significant differences were found between methods. Estimated rates of realized genetic gain ranged from 0.19 to 0.32 in genetic standard deviation units. The error of the various methods was also highly variable and was considered an important factor when comparing the efficiency of different methods because error indicates how close the estimates are to the true values of realized genetic gain. Based on these evaluation criteria, the best methods for the accurate estimation of genetic gain were the estimated breeding value, control population, and era trial method. The EBV method was best in terms of performance, feasibility, and cost, but it requires the application of a good control strategy in trials and the keeping of complete pedigree records right from the start of the breeding program. However, no single analytical method is suitable for all situations. The selection of a statistical method should be guided by resources and the structure of the available breeding program data (Covarrubias-Pazaran 2020; Rutkoski 2019a).

### Current Limitations

Thus, as shown above, the literature on genetic gain in rice is rich. Despite this diversity, certain aspects of genetic gain assessment by rice breeding programs have received little attention. Below, we present the elements that we consider potentially important for obtaining more accurate estimates of genetic gain and facilitating comparisons between studies.

#### Benchmark for Comparing Gains (Absolute vs. Relative Gain)

Genetic gain is often reported in terms of phenotypic units per cycle or year (absolute) or as a percentage relative to a baseline. The rate of genetic gain per year is considered the best estimate for comparing breeding strategies, which may differ in terms of the number of years per cycle (Hallauer et al. 2010). However, this relative gain depends strongly on the baseline and, therefore, on the estimation method. Indeed, as highlighted here, authors use different baselines to calculate the relative genetic gain and do not even specify the baseline used in some cases (dos Reis et al. 2015; Tabien et al. 2008). Relative genetic gain, as a percentage, is generally estimated as the ratio of the slope to the intercept, with the intercept corresponding to the start of the breeding program (Breseghello et al. 2011; da Costa et al. 2021; Silva Júnior et al. 2021). However, in some cases, it is estimated relative to the performance of the first variety released (Peng et al. 2000). Caution is required when drawing conclusions about the results for relative genetic gain because the results of the calculation depend strongly on the baseline used. The use of more recent varieties as a baseline results in lower relative genetic gain values than the use of older varieties (Ahrends et al. 2018). The benchmark issue also arises in studies providing multiple estimates of gain by ecosystem, population type, or year. Inverse trends are often observed for absolute and relative gain values (Kumar et al. 2021; Peng et al. 2000; Venkatanagappa et al. 2021), clearly demonstrating the difficulty of selecting an appropriate genetic gain reference for comparison between studies. We, therefore, recommend that readers pay attention to this limitation when trying to compare studies. This is a key aspect for improving the accuracy of genetic gain comparisons in terms of performance and for the potential optimization of rice breeding programs.

#### Connectivity Between Cycles or Years

The main limitation of using historical data to estimate the realized rate of genetic gain, whether for grain yield or other traits of interest, is the lack of connectivity between experiments. Most studies based on historical data incorporate controls into the evaluation process, but detailed information about the control strategy is often lacking. A variable control strategy, in which the controls are progressively replaced with newly released cultivars over time, is used in almost all historical studies. However, the frequency and intensity of control replacement are not specified in most studies (Breseghello et al. 2011; dos Reis et al. 2015; DoVale et al. 2012; Juma et al. 2021; Morais Júnior et al. 2015; Silva Júnior et al. 2021; Soares et al. 1999; Streck et al. 2018b). Conversely, a few authors have described well the connectivity of the historical data used through a variable control strategy (renewal frequency of 10 years on average). On average, five controls (common cultivars) in each experiment were evaluated in consecutive years and eventually replaced by recently released cultivars and/or cultivars from other collaborators' programs (da Costa et al. 2021). Khanna et al. (2022) also highlighted the connectivity of their dataset through the use of long-term checks, the re-evaluation of superior genotypes in successive years, and the incorporation of the relationship matrix based on pedigree into the model. This second strategy is almost never used to control for connectivity. Four of the 31 studies presented here used pedigree information to increase connectivity to obtain better estimates of genetic value (Biswas et al. 2023; Juma et al. 2021; Khanna et al. 2022, 2023). Connectivity is important for the accuracy of genetic gain estimates, and taking this factor into account can improve the separation of genetic and non-genetic effects (environment, agronomic practice, etc.).

#### Coverage of Target Environments

The number of environments covered by the study (seasons, years, and/or locations) is also a key factor for obtaining relevant estimates. Coverage of the target environments is greater with historical data, which are mostly obtained in multi-environment trials. Discussions about target environment coverage, therefore, arise principally in the context of era studies. Indeed, the use of a replicated design for the evaluation of cultivars released over a period of time improves the monitoring of genotype-by-environment interactions, thereby providing more accurate estimates. However, most of the era studies reviewed here covered a relatively small number of environments. They were frequently performed at single-site stations over one to four cropping seasons (Peng et al. 2000; Souza et al. 2007; Tabien et al. 2008). Results for similar numbers of trials have been reported for other cereals (Duvick 1984; Hanif et al. 2022; Xiao et al. 2012; Yadav et al. 2021). It is important to conduct era trials across a well-defined target population of environments (TPEs) over many years to obtain a more accurate evaluation of the genetic gain of varieties, but this is both resource- and labor-intensive, limiting the possibilities for such an approach, particularly in conditions in which research funds are limiting. This limitation decreases the attractiveness of this method relative to historical data, for which no such additional investment is required. Nevertheless, the TPE coverage of era trials can be improved by adapting one of the following strategy trials, as demonstrated in other crop species. In sunflower, the genetic progress in oil yield was estimated from 122 on-farm trials of commercial and near-commercial sunflower hybrids across 32 sites in central Argentina (de la Vega et al. 2007). Moreover, on-farm trials should provide a more accurate assessment of genetic gain in farmers' fields because, to our knowledge, no studies have been performed to assess the on-farm genetic gain for rice grain.

#### Non-genetic Trend Evaluation

Both the genetic improvement of newly released varieties and agronomic practices (fertilizer, plant protection, tillage, weed control) may contribute to increases in grain yields. In experimental studies, the effect of the environment or, more generally, non-genetic effects may bias the estimation of the genetic effect. Most of the rice studies presented here addressed this issue implicitly by providing estimates of genetic gain incorporating the contributions of these major factors. Only two of the studies considered reported non-genetic trends explicitly (Kumar et al. 2021; Rahman et al. 2023). In their study, Kumar et al. (2021) evaluated the proportion of grain yield increase due to genetic factors in a rainfed environment prone to drought. Their findings indicated the yield increase was primarily due to genetic factors rather than non-genetic factors, regardless of the stress level. For Rahman et al. (2023), a large proportion of the gain in grain yield in Bangladesh over the last 50 years was due to non-genetic factors. Therefore, it is important to determine the genetic contribution of newly released varieties to the total yield trend, to gain a better appreciation of the contribution of breeding to the improvement of production. Several studies on cereals, such as wheat, maize, barley, oat, and on other crops (sugar beet, ryegrass, rapeseed, etc.) have dissected the genetic and environmental contributions to yield trends (Bornhofen et al. 2018; Laidig et al. 2014; Mackay et al. 2011; Piepho et al. 2014; Schuster 1997). Some of these studies have pointed out the importance of considering the effect of diseases (the breakdown of disease resistance for older genotypes), which may bias the estimation of long-term genetic and non-genetic effects, potentially leading to an overestimation of genetic trends based on long-term yield trial data (Mackay et al. 2011; Piepho et al. 2014).

#### Sampling of Genetic Variance

The method for sampling the genotypes to be evaluated was not explicitly explained in most of the era studies. The set of genotypes used appeared to represent all the varieties developed by the program (provided that there is enough viable seed stock available). This would also explain the size of the era panels, which were relatively small (less than 50 genotypes for seven out of nine era studies) and spanning a period of up to 60 years of selection. The management and conservation of germplasm in public sector breeding programs is challenging. Nevertheless, a few studies have described rationales for the sample of cultivars for evaluation. In the study by Peng et al. (2000), the 12 genotypes evaluated in the era trial included 10 released cultivars selected on the basis of the cultivated area they occupied during different historical periods. The other two genotypes were control breeding lines included based on their high performance in yield trials. The rationale underlying the composition of the era panel was also reported in other genetic gain studies in wheat and maize. Masuka et al. (2017) used an era panel composed of maize hybrids selected on the basis of their superior performance in regional trials. The era panel may be selected based on the popularity of varieties released by the breeding program. In this case, however, due to the lack of adaptation of certain varieties by farmers, the final panel may be too small for an accurate assessment of genetic gain. However, the sampling of the most advanced lines (including released varieties) of each cycle rather than just released varieties is recommended to increase the representativeness of the genetic material included in the program, thereby improving the accuracy of genetic gain estimates.

#### Rice Hybrids

There is a significant lack of assessment of genetic gain achieved by hybrid rice breeding programs. A single study on genetic gain incorporating hybrids was conducted by Venkatanagappa et al. (2021). The study used five hybrid varieties in combination with 39 inbred varieties, making it challenging to evaluate the impact of hybrids on genetic gain. Apart from this study, two other studies have reported the evaluation of genetic progress in hybrids. In India, Muralidharan et al. (2022), reported that the evolution of grain yield in F1 hybrids evaluated in irrigated ecosystems with four maturity groups did not show any significant progress over a period of 32 years of hybrid breeding. They concluded that F1 hybrids only resulted in a 10% increase in grain yield compared to inbred cultivars in less than 20% of the testing locations. According to a study by Zhu et al. (2016) in China, five hybrid varieties were evaluated in combination with nine inbred varieties from several companies. In this study, the hybrids were more recent compared to inbred varieties, but the authors did not draw any conclusions on the contribution of the hybrids to the grain yield progress. It is, therefore, challenging to identify a pattern of genetic gain on grain yield or compare the genetic gains achieved in rice hybrid breeding due to the limited available data on the subject. In the upcoming years, there should be a greater effort in reporting the realized genetic gain on hybrids by the rice hybrid breeding programs to evaluate the impact of this technology.

#### Gain Per Unit Cost

The efficiency of plant breeding programs is usually evaluated by estimating the rate of genetic gain per unit of time. However, the breeders’ ability to maximize the rate of genetic gain is constrained by limited resources and time. Plant breeders must, therefore, take into account multiple constraints if they are to maximize genetic gain, and the ideal trade-off between genetic gain per unit cost and the maximal rate of genetic gain is not always obvious. Most of the studies reviewed here drew conclusions about the efficiency of their programs based on positive rates of genetic gain, but none actually considered costs in the analysis. This would not, in any case, be feasible for studies covering a very long period or combining information from several programs. However, the integration of costs is relevant when the objective is to evaluate a specific breeding program or the impact of new breeding techniques (Barros et al. 2018; Morais Júnior et al. 2017). For this reason, cost integration is currently performed mostly in simulation studies. Atlin and Econopouly (2021) showed that despite the increase in gain with population size and selection intensity, gains were optimal, in terms of the cost per unit gain, for relatively small population sizes and moderate selection intensities. We advise readers to consider the cost per unit genetic gain when possible, as a means of assessing the efficiency of their programs or for comparing alternative breeding strategies, because higher rates of genetic gain may not necessarily be economically efficient.

## Implications for Breeding

### Recommendations for Increasing Genetic Gain

**Fig. 5:**
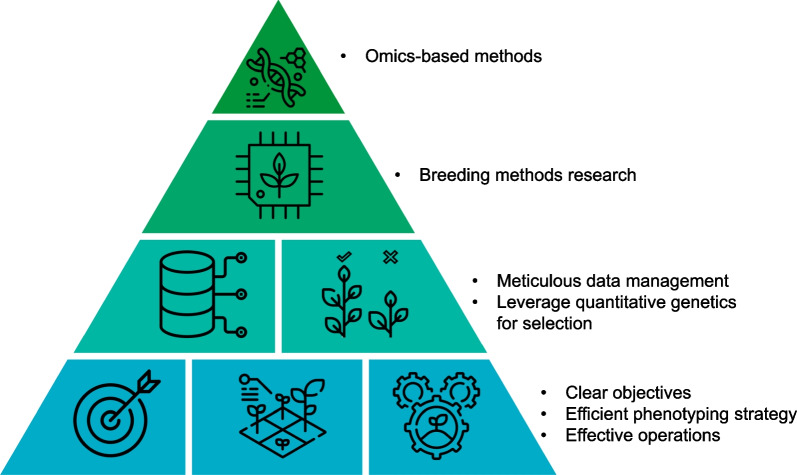
Hierarchy of the components of a breeding program for achieving genetic gain. The elements at the base of the pyramid are the fundamental components on which the program is based to deliver genetic gain. The elements at the top of the pyramid are advanced components allowing optimization of the program (adapted from Rutkoski 2019a)

As indicated above, the rate of genetic gain varies considerably between studies, and, with a mean value of 0.92%, there is room for improvement. Each breeding program has its specific features, but they are all based on the same main components. Rutkoski (2019a) presented the hierarchy of components required to achieve the targeted genetic gains (Fig. 5). Each component can be improved, ultimately increasing the rate of genetic gain for target traits. Below, we highlight the relevant breeding strategy elements in the context of rice breeding.

#### Clear Objectives

An absence of clear objectives (or ideotypes) was identified as a major driver of low genetic gain in the initial phases of establishment in several rice breeding programs (Breseghello et al. 2011; da Costa et al. 2021; Streck et al. 2018b). The objectives of a breeding program are usually defined by the breeders and not explicitly described. The reference ideotype is often defined based on the most popular variety in a given market. This approach has led to significant progress, but the correspondence of the products of the program and the needs of farmers and end-users may be limited, due to changes in the context over time. The expectations of the sector (grain quality, earliness, etc.) may change, as may agroclimatic conditions (pathogens, abiotic stress). This issue can be addressed, to achieve the desired level of genetic gain for the target traits, by developing a product concept based on the needs of the rice sector and translating it into breeding objectives. The product concept describes the target attributes of the products (varieties) for a specific sector of the market on which breeding efforts are focused (Cobb et al. 2019). One effective way to use this information in breeding programs involves defining an index of selection with appropriate economic weights, because target traits may differ in terms of their genetic variance, heritability or economic importance (Hazel et al. 1994).

#### Data Management

The selection of the best candidates is based essentially on pedigree information and data collected during the phenotyping stages. Phenotyping represents a large part of the investment in breeding programs, and ensuring the quality and traceability of the pedigree and phenotypic data is challenging in all breeding programs. Data management systems have been developed to assist breeders with these tasks. However, few public breeding programs currently use these tools, despite the crucial nature of data quality for breeding processes, because errors and data loss decrease selection accuracy, thereby also decreasing genetic gain (Rutkoski 2019a). The use of tools for digitized data collection, and for the management and sharing of breeding data is essential, to ensure high data quality, selection accuracy, and improvements in genetic gain (Breseghello et al. 2021). Moreover, if well managed, historical datasets from breeding programs (phenotypic, genotypic, and pedigree data) can be repurposed to address other components of the breeding strategy. For example, data from several years of multi-environment trials can be used to investigate genotype-by-environment interactions, making it possible to improve the definition of the target populations of environments (Breseghello et al. 2021; Covarrubias-Pazaran 2020). Historical data can also facilitate the implementation of genomic selection, as pedigree and marker data can initially be combined to improve prediction accuracy (Legarra et al. 2014).

#### Recurrent Selection Based on Elite Material

As a model species and a major crop, rice has been intensively investigated, to characterize its genetic diversity (Wang et al. 2018) and the genetic architecture of its agronomic traits (Miura et al. 2011). Several dozen genes or QTLs with large effects on phenotype have been detected, particularly for biotic stress resistance. This fascination with QTLs has blurred the line between breeding and pre-breeding stages. Indeed, in relation to the concept of “breeding by design” (Peleman and van der Voort 2003), approaches have been developed to “exploit grain yield genes” in breeding programs (Sakamoto and Matsuoka 2008; Xing and Zhang 2010). Even though yield components have been well characterized in rice, little progress has been made with this approach, as yield is a quantitative trait highly prone to genotype-by-environment interactions. For the consistent improvement of quantitative traits, such as yield, there needs to be a clear separation between pre-breeding and breeding. Pre-breeding activities should focus on the introgression of favorable alleles into the elite germplasm. This enhancement of the germplasm may be performed through the deployment of QTLs/alleles/haplotypes in the elite gene pool or, in some cases, in the final product, by introgression, without contaminating the elite gene pool with parents of lower breeding value (Cobb et al. 2019). A good characterization of the elite germplasm is therefore required to determine its variability and the frequency of the favorable alleles for the major genes. This has been done, for example, at IRRI, where elite lines representing the diversity of the breeding programs were selected based on breeding values for grain yield and characterized for resistance to major diseases or stresses (Juma et al. 2021; Khanna et al. 2022). During breeding activities, population improvement through recurrent selection should be implemented, to increase the frequency of favorable alleles for quantitative traits, to ensure long-term genetic gain. The effectiveness of closed recurrent selection strategies for achieving genetic gain, maintaining genetic variability, and increasing the potential for selection of superior lines was highlighted in Embrapa’s irrigated and upland rice breeding programs (Barros et al. 2018; Breseghello et al. 2009; Morais Júnior et al. 2015, 2017).

#### Shortening the Breeding Cycle

Reducing the time required to complete a breeding cycle (recycling advanced material as parents) is one of the most efficient methods for increasing the rate of genetic gain (Atlin and Econopouly 2021; Cobb et al. 2019). Several techniques, such as off-season nurseries, early testing, and rapid generation advances (RGA) have been used over the years to reduce breeding cycle length in rice breeding programs. Depending on the objective and constraints of the breeding program, one or more of these techniques can be used to reduce the length of the breeding cycle. Typically, without optimization, the breeding cycle lasts eight to 10 years. The integration of these techniques can significantly reduce that duration. For example with RGA, breeders have been able to reduce breeding cycle length by at least two years (Collard et al. 2017, 2019; Lenaerts et al. 2018; Tanaka et al. 2016). When RGA is optimized, line fixation from F2 to F6 takes only one year. More recently, genomic selection (GS) has emerged as the most powerful tool yet for reducing cycle length. GS is based on the use of a model to predict genetic value from genome-wide marker loci, followed by selection based on the predicted values (Meuwissen et al. 2001). With genomic selection, the breeding cycle may be reduced to almost a year, as the only requirement for predicting the performance of selection candidates is genotyping data. In rice, GS has been increasingly explored in breeding programs over the last decade, which has rendered its application more efficient with respect to selection objectives (Bartholomé et al. 2022). As an advanced tool, genomic selection should be considered in breeding programs in which all the other components are already in place (Fig. 5).

### Use of Computer Simulations for Optimizing Breeding Strategies

The performance of a breeding program or of the integration of new breeding techniques is commonly assessed by a posteriori estimation of the realized genetic gain. However, given the complexity of breeding schemes and the cost of implementing multiple experiments, computer simulations are increasingly used for rapid, cost-effective evaluations of a wide range of scenarios (Sun et al. 2011). Here, we discuss the use of genetic gain and the usefulness of computer simulation in the design and strategic optimization of breeding programs. Computer simulation models are of two types: deterministic simulation and stochastic simulation models. Deterministic simulation uses equations based on quantitative genetics principles to predict the response to selection from knowledge of population characteristics (selection intensity, heritability, selection accuracy). However, it is difficult to incorporate certain breeding operations, including crossing design, generation advancement, use of new genetics. Deterministic simulations are therefore complex to implement in plant breeding and are more approximate than stochastic stimulation. Few studies in plant breeding are based on deterministic simulation. One example is the development of deterministic modeling based on the breeder's equation, including operating costs, to guide breeding pipeline design (Atlin and Econopouly 2021). Simulation results have shown that decreasing the length of the breeding cycle is a more cost-effective method of increasing genetic gain than increasing population size and selection intensity.

Stochastic simulations generate genotypic and phenotypic data for each simulated individual, which are then used in the traditional steps of a breeding program (crossing, evaluation and selection) (Li et al. 2012; Phocas 2011). Simulation tools can be used in prospective studies: i) to evaluate the performance of breeding strategies in the medium and long term; ii) to compare several strategies and to guide decisions; iii) to identify the most effective breeding strategies. As suggested by their name, stochastic simulations have a random component and several replicates are, therefore, required, ultimately resulting in long computation times (Li et al. 2012; Phocas 2011). Stochastic simulation is also relatively simple but requires more computational resources to simulate more complex breeding programs. In rice, a few studies have been performed with stochastic simulation, to evaluate the efficiency of breeding programs and to optimize these programs. With the objective of optimizing QTL introgression, (Platten and Fritsche-Neto 2022) compared three strategies for developing new recipients for QTL introgression (background recovery, selective sweep, and breeding values) in a short-term rice breeding program through stochastic simulations performed with the AlphaSimR package (Gaynor et al. 2021). They showed that the breeding value strategy with 10 selected parents gave the best trade-off between a lower penalty for introducing new QTLs and the fixation of these QTLs at a reasonable speed over subsequent breeding cycles, based on the population mean performance. Rutkoski (2019b) conducted stochastic simulations of eight rice breeding scheme scenarios to compare the efficiency of five methods for estimating genetic gain in terms of error, precision, efficiency, and correlation between true breeding values and predicted breeding values. In this study, the effects of trait heritability and breeding cycle length on realized genetic gain were also evaluated.

Studies on the optimization of breeding programs through computer simulation have been conducted in other cereal species, to increase the rate of genetic gain for yield. These studies have included comparisons between new selection strategies to identify the best strategy, evaluation of the efficiency of genomic selection for increasing the rate of genetic gain, and the use of new crossing methods, for example. Simulations have also been used to investigate various aspects in genomic selection (GS) optimization studies, including the robustness of statistical models, comparisons of alternative GS breeding schemes and assessments of the impact of GS on long-term genetic gain and inbreeding, resource allocation, training population structure, and the updating of models on the maximization of prediction accuracy and genetic gain (Bastiaansen et al. 2012; Daetwyler et al. 2010; Lorenz 2013; Muleta et al. 2019; Müller et al. 2017). All these simulations can be adapted to rice, to optimize breeding programs and increase the rate of genetic gain for yield and other agronomic traits by making use of the development of ever more powerful, accessible and easy-to-use simulation tools, and to simulate complex breeding programs integrating biotechnologies (Gaynor et al. 2021; Liu et al. 2019; Pook et al. 2020).

## Conclusion

The realized genetic gain achieved by a breeding program is a key indicator of its effectiveness. Increasing numbers of studies over the last decade have focused on genetic gain for grain yield and other important agronomic traits in rice breeding programs, highlighting the interest of rice breeders in monitoring the impact of the decisions more effectively. Genetic gain for grain yield varied considerably between studies. Estimates are difficult to compare directly between studies, due to differences in the estimation method, source of data, populations evaluated or environmental factors. However, it is clear that significant rates of genetic gain can be achieved (greater than 1.5%). Based on a review of the various studies, we highlight the main points on which breeding programs should focus: i) defining clear breeding objectives based on a product concept, ii) use of a data management system to reduce errors and increase the reuse of data, iii) clear separation of breeding and pre-breeding activities to focus on the improvement of elite germplasm and iv) achieving the right balance between cycle length and evaluation steps. The accuracy of genetic gain estimates was most commonly limited by the restricted use of pedigree data, a lack of evaluation of non-genetic trends, or a lack of information regarding the statistical method used for estimation. Improving these elements in future studies should be straightforward, thereby facilitating comparisons.

### Supplementary Information

**Additional file 1: Table S1**. Summary of studies on genetic gain for yield of rice breeding programs; **Table S2**. Summary of studies on genetic gain for days to heading, plant height, grain quality and disease resistance in rice breeding programs.

## Data Availability

The datasets analyzed in this study are included in this published article and its additional files.

## Abbreviations

BLUE: Best linear unbiased estimator
BLUP: Best linear unbiased predictor
EBV: Estimated breeding value
GS: Genomic selection
GxE: Genotype-by-environment interaction
QTLs: Quantitative trait loci
RGA: Rapid generation advance
TPE: Target population of environments

## References

[CR1] Abidine Fellahi ZE, Hannachi A, Bouzerzour H, Abidine Fellahi ZE, Hannachi A, Bouzerzour H (2020). Expected genetic gains from mono trait and index-based selection in advanced bread wheat (*Triticum aestivum* L.) populations. Revista Facultad Nacional De Agronomía Medellín.

[CR2] Acquaah G (2009). Principles of plant genetics and breeding.

[CR3] Ahrends HE, Eugster W, Gaiser T, Rueda-Ayala V, Hüging H, Ewert F, Siebert S (2018). Genetic yield gains of winter wheat in Germany over more than 100 years (1895–2007) under contrasting fertilizer applications. Environ Res Lett.

[CR4] Allard RW (1999). Principles of plant breeding.

[CR5] Alves NB, Balestre M, Pennacchi JP, Nunes Fernandes MC, Goulart Castro D, Barbosa Silva Botelho F (2020). Genetic progress of upland rice (*Oryza sativa* L.) lines for disease resistance. Plant Breed.

[CR6] Atlin GN, Econopouly BF (2021). Simple deterministic modeling can guide the design of breeding pipelines for self-pollinated crops. Crop Sci.

[CR7] Baker RJ (1984) Quantitative genetic principles in plant breeding. In: Gustafson JP (Ed) Gene manipulation in plant improvement: 16th Stadler genetics symposium, pp 147–176. Springer, US. 10.1007/978-1-4613-2429-4_7

[CR8] Barros MS, Morais Júnior OP, Melo PGS, Morais OP, Castro AP, Breseghello F (2018). Effectiveness of early-generation testing applied to upland rice breeding. Euphytica.

[CR9] Bartholomé J, Prakash PT, Cobb JN (2022) Genomic prediction: progress and perspectives for ricerice improvement. In: Ahmadi N, Bartholomé J (Eds) Complex trait prediction: methods and protocols, pp 569–617. Springer, US. 10.1007/978-1-0716-2205-6_21

[CR10] Bastiaansen JW, Coster A, Calus MP, van Arendonk JA, Bovenhuis H (2012). Long-term response to genomic selection: effects of estimation method and reference population structure for different genetic architectures. Genet Select Evolut GSE.

[CR11] Biswas PS, Ahmed MME, Afrin W, Rahman A, Shalahuddin AKM, Islam R, Akter F, Syed MA, Sarker MRA, Ifterkharuddaula KM, Islam MR (2023). Enhancing genetic gain through the application of genomic selection in developing irrigated rice for the favorable ecosystem in Bangladesh. Front Genet.

[CR12] Bornhofen E, Todeschini MH, Stoco MG, Madureira A, Marchioro VS, Storck L, Benin G (2018). Wheat yield improvements in Brazil: roles of genetics and environment. Crop Sci.

[CR13] Bouffier L, Raffin A, Kremer A (2008). Evolution of genetic variation for selected traits in successive breeding populations of maritime pine. Heredity.

[CR14] Breseghello F, Rangel PHN, de Morais OP (1999). Yield gain through irrigated rice breeding in the northeast Brazil. Pesq Agrop Brasileira.

[CR15] Breseghello F, Morais O, Castro E, Prabhu A, Bassinello P, Pereira J, Utumi M, Ferreira M, Soares A (2009). Recurrent selection resulted in rapid genetic gain for upland rice in Brazil. Int Rice Res Notes.

[CR16] Breseghello F, Mello RN, Pinheiro PV, Soares DM, Lopes Júnior S, Nakano Rangel PH, Guimarães EP, Castro AP, Colombari Filho JM, Magalhães Júnior AM, Fagundes PRR, Neves CF, P., Furtini, I. V., Utumi, M. M., Pereira, J. A., Cordeiro, A. C. C., Filho, A. S., Abreu, G. B., Moura Neto, F. P., … Crossa, J.  (2021). Building the Embrapa rice breeding dataset for efficient data reuse. Crop Sci.

[CR17] Breseghello F, de Morais OP, Pinheiro PV, Silva ACS, da Maia de Castro E, Guimarães ÉP, de Castro AP, Pereira JA, de Matoslopes A, Utumi MM, de Oliveira JP (2011) Results of 25 Years of upland rice breeding in Brazil. Crop Sci 51(3):914. 10.2135/cropsci2010.06.0325

[CR18] Briggs WH, Goldman IL (2006). Genetic variation and selection response in model breeding populations of Brassica rapa following a diversity bottleneck. Genetics.

[CR19] Bulmer MG (1971). The effect of selection on genetic variability. Am Nat.

[CR20] Bulmer MG (1976). The effect of selection on genetic variability: a simulation study. Genet Res.

[CR21] Ceccarelli S (2015). Efficiency of plant breeding. Crop Sci.

[CR22] Chauhan BS, Jabran K, Mahajan G (2017). Rice Production worldwide.

[CR23] Cobb JN, Biswas PS, Platten JD (2018). Back to the future: revisiting MAS as a tool for modern plant breeding. TAG Theor Appl Genet.

[CR24] Cobb JN, Juma RU, Biswas PS, Arbelaez JD, Rutkoski J, Atlin G, Hagen T, Quinn M, Ng EH (2019). Enhancing the rate of genetic gain in public-sector plant breeding programs: lessons from the breeder’s equation. Theor Appl Genet.

[CR25] Collard BCY, Beredo JC, Lenaerts B, Mendoza R, Santelices R, Lopena V, Verdeprado H, Raghavan C, Gregorio GB, Vial L, Demont M, Biswas PS, Iftekharuddaula KM, Rahman MA, Cobb JN, Islam MR (2017). Revisiting rice breeding methods—evaluating the use of rapid generation advance (RGA) for routine rice breeding. Plant Prod Sci.

[CR26] Collard BCY, Gregorio GB, Thomson MJ, Islam MR, Vergara GV, Laborte AG, Nissila E, Kretzschmar T, Cobb JN (2019). Transforming Rice breeding: re-designing the irrigated breeding pipeline at the International Rice Research Institute (IRRI). Crop Breed Genet Genom.

[CR27] Covarrubias-Pazaran G (2020) EiB-M2 Breeding process assessment-Genetic Gain. https://excellenceinbreeding.org/sites/default/files/manual/EiB-M2_Breeding%20process%20assessment-Genetic%20Gain_20-11-20.pdf

[CR28] Crossa J, Pérez-Rodríguez P, Cuevas J, Montesinos-López O, Jarquín D, de los Campos, G., Burgueño, J., González-Camacho, J. M., Pérez-Elizalde, S., Beyene, Y., Dreisigacker, S., Singh, R., Zhang, X., Gowda, M., Roorkiwal, M., Rutkoski, J., & Varshney, R. K.  (2017). Genomic selection in plant breeding: methods, models, and perspectives. Trends Plant Sci.

[CR29] Cruz M, Arbelaez JD, Loaiza K, Cuasquer J, Rosas J, Graterol E (2021). Genetic and phenotypic characterization of rice grain quality traits to define research strategies for improving rice milling, appearance, and cooking qualities in Latin America and the Caribbean. Plant Genome.

[CR30] da Costa WG, Silva Júnior AC, Barbosa IP, Cruz CD, Borém A, Soares PC, Gonçalves RP, Torga PP, Condé ABT (2021). Quarter century genetic progress in irrigated rice (*Oryza sativa*) in Southeast Brazil. Plant Breed.

[CR31] da Silva Júnior AC, Carneiro VQ, dos Santos IG, Rosado RDS, Cruz CD, Soares PC (2021). Genetic progress over twenty-three years of irrigated rice breeding in southeastern Brazil. Acta Sci Agron.

[CR32] Daetwyler HD, Pong-Wong R, Villanueva B, Woolliams JA (2010). The impact of genetic architecture on genome-wide evaluation methods. Genetics.

[CR33] de la Vega AJ, DeLacy IH, Chapman SC (2007). Progress over 20 years of sunflower breeding in central Argentina. Field Crop Res.

[CR34] de Morais Júnior OP, Melo PGS, de Morais OP, de Castro AP, Breseghello F, Utumi MM, Pereira JA, Wruck FJ, Colombari Filho JM (2015). Genetic progress after cycles of upland rice recurrent selection. Scientia Agricola.

[CR35] Dingkuhn M, Laza MRC, Kumar U, Méndez KVS, Collard B, Jagadish KSV, Singh RK, Padolina T, Malabayabas M, Torres Castro EA, Rebolledo MC, Manneh B, Sow A (2015). Improving yield potential of tropical rice: achieved levels and perspectives through improved ideotypes. Field Crop Res.

[CR36] dos Reis GG, dos, Fritsche-Neto, R., Soares, P. C., CornÃ©lio, V. M. de O., Reis, M. de S., Morais, O. P. de, & Marques, T. da S.  (2015). Accuracy and genetic progress of agronomic traits in irrigated rice program in Brazil. Afr J Agric Res.

[CR37] DoVale JC, Soares P, Cornélio V, Reis M, Borges V, Barcelos Bisi R, Soares A, Fritsche-Neto R (2012). Genetic contribution in yield of irrigated rice in Minas Gerais State between 1998 and 2010. Bragantia.

[CR38] Dudley JW (2007). From means to QTL: the Illinois Long-Term selection experiment as a case study in quantitative genetics. Crop Sci.

[CR39] Dudley J (1997) Quantitative genetics and plant breeding. 10.1016/S0065-2113(08)60051-6

[CR40] Duvick DN (1984) Genetic contributions to yield gains of U.S. hybrid maize, 1930 to 1980. In: Genetic contributions to yield gains of five major crop plants, pp 15–47. John Wiley & Sons, Ltd. 10.2135/cssaspecpub7.c2

[CR41] Duvick DN (2005) The contribution of breeding to yield advances in maize (Zea mays L.). In: Advances in agronomy, Vol. 86, pp 83–145. Academic Press. 10.1016/S0065-2113(05)86002-X

[CR42] Eberhart SA (1964). Least squares method for comparing progress among recurrent selection methods 1. Crop Sci.

[CR43] Falconer DS (1981) Introduction to quantitative genetics (Second edition). Longman Group Limited. London and New York

[CR44] FAO, F. and A. O. of U. N (2022) FAOSTAT. https://www.fao.org/faostat/en/#data/QCL

[CR45] Feng F, Li Y, Qin X, Liao Y, Siddique KHM (2017). Changes in Rice Grain Quality of Indica and Japonica Type Varieties Released in China from 2000 to 2014. Front Plant Sci.

[CR46] Fischer RA, Edmeades GO (2010). Breeding and cereal yield progress. Crop Sci.

[CR47] Gallais A (2011) Méthodes de création de variétés en amélioration des plantes. Editions Quae

[CR48] Garrick DJ (2010). An animal breeding approach to the estimation of genetic and environmental trends from field populations1. J Anim Sci.

[CR49] Gaynor RC, Gorjanc G, Hickey JM (2021). AlphaSimR: an R package for breeding program simulations. G3 Genes Genomes Genet.

[CR50] GRiSP (2013) Rice Almanac, 4th Edition. http://archive.org/details/RiceAlmanac

[CR51] Hallauer AR, Carena MJ, Filho JBM (2010). Quantitative genetics in maize breeding.

[CR52] Hanif U, Gul A, Amir R, Munir F, Sorrells ME, Gauch HG, Mahmood Z, Subhani A, Imtiaz M, Alipour H, Rasheed A, He Z (2022). Genetic gain and G×E interaction in bread wheat cultivars representing 105 years of breeding in Pakistan. Crop Sci.

[CR53] Hazel LN, Dickerson GE, Freeman AE (1994). The selection index—then, now, and for the future. J Dairy Sci.

[CR54] Heffner EL, Lorenz AJ, Jannink J-L, Sorrells ME (2010). Plant breeding with genomic selection: gain per unit time and cost. Crop Sci.

[CR55] Helms TC, Hammond JJ (2006). Genetic gain equation with correlated genotype × environment effects. Crop Sci.

[CR56] Huehn M (2005). Optimum number of crosses and progeny per cross in breeding self-fertilizing crops. II. Numerical results based on expected selection responses (special case). Cereal Res Commun.

[CR57] Juma RU, Bartholomé J, Thathapalli Prakash P, Hussain W, Platten JD, Lopena V, Verdeprado H, Murori R, Ndayiragije A, Katiyar SK, Islam MR, Biswas PS, Rutkoski JE, Arbelaez JD, Mbute FN, Miano DW, Cobb JN (2021). Identification of an Elite Core panel as a key breeding resource to accelerate the rate of genetic improvement for irrigated rice. Rice.

[CR58] Khanna A, Anumalla M, Catolos M, Bartholomé J, Fritsche-Neto R, Platten JD, Pisano DJ, Gulles A, Sta. Cruz, M. T., Ramos, J., Faustino, G., Bhosale, S., & Hussain, W.  (2022). Genetic trends estimation in irris rice drought breeding program and identification of high yielding drought-tolerant lines. Rice.

[CR59] Khanna A, Ramos J, Cruz MTS, Catolos M, Anumalla M, Godwin A, Gregorio G, Singh RK, Dixit S, Ali J, Islam MR, Singh VK, Rahman A, Khatun H, Pisano DJ, Bhosale S, Hussain W (2023) Genetic gains in IRRI’s rice salinity breeding and elite panel development as a future breeding resource, p 2023.06.14.544895. bioRxiv. 10.1101/2023.06.14.544895

[CR60] Khush G, Virk P (2005) IR varieties and their impact. Los Baños (Philippines): International Rice Research Institute

[CR61] Khush GS (2008) Historical review of rice breeding and the future prospects. 18

[CR62] Kim H-Y, Ko J, Kang S, Tenhunen J (2013). Impacts of climate change on paddy rice yield in a temperate climate. Glob Change Biol.

[CR63] Kumar A, Raman A, Yadav S, Verulkar SB, Mandal NP, Singh ON, Swain P, Ram T, Badri J, Dwivedi JL, Das SP, Singh SK, Singh SP, Kumar S, Jain A, Chandrababu R, Robin S, Shashidhar HE, Hittalmani S, Piepho HP (2021). Genetic gain for rice yield in rainfed environments in India. Field Crops Res.

[CR64] Laidig F, Piepho H-P, Drobek T, Meyer U (2014). Genetic and non-genetic long-term trends of 12 different crops in German official variety performance trials and on-farm yield trends. Theor Appl Genet.

[CR65] Legarra A, Christensen OF, Aguilar I, Misztal I (2014). Single step, a general approach for genomic selection. Livest Sci.

[CR66] Lenaerts B, de Mey Y, Demont M (2018). Global impact of accelerated plant breeding: evidence from a meta-analysis on rice breeding. PLoS ONE.

[CR67] Li X, Zhu C, Wang J, Yu J (2012) Chapter six—computer simulation in plant breeding. In: Sparks DL (Ed) Advances in agronomy, Vol 116, pp 219–264. Academic Press. 10.1016/B978-0-12-394277-7.00006-3

[CR68] Liu F, Wang P, Zhang X, Li X, Yan X, Fu D, Wu G (2018). The genetic and molecular basis of crop height based on a rice model. Planta.

[CR69] Liu H, Tessema BB, Jensen J, Cericola F, Andersen JR, Sørensen AC (2019). ADAM-plant: a software for stochastic simulations of plant breeding from molecular to phenotypic level and from simple selection to complex speed breeding programs. Front Plant Sci.

[CR70] Liu M, Tong H, Liu Y, Li C, Wu X, Li M, Li X, Tang Y (2021). Genetic progress in grain yield and the associated physiological traits of popular wheat in southwestern China from 1969 to 2012. Crop Sci.

[CR71] Lorenz AJ (2013). Resource allocation for maximizing prediction accuracy and genetic gain of genomic selection in plant breeding: a simulation experiment. G3 Genes Genomes Genetics.

[CR72] Luckett D, Halloran G (2017) Plant breeding. In: Pratley J (Ed), Principles of field crop production (4th ed.). Graham Centre for Agricultural Innovation

[CR73] Mackay I, Horwell A, Garner J, White J, McKee J, Philpott H (2011). Reanalyses of the historical series of UK variety trials to quantify the contributions of genetic and environmental factors to trends and variability in yield over time. Theor Appl Genet.

[CR74] Mackill DJ (2018). Special issue: iconic rice varieties. Rice.

[CR75] Mackill DJ, Khush GS (2018). IR64: a high-quality and high-yielding mega variety. Rice.

[CR76] Masuka B, Atlin GN, Olsen M, Magorokosho C, Labuschagne M, Crossa J, Bänziger M, Pixley KV, Vivek BS, von Biljon A, Macrobert J, Alvarado G, Prasanna B, m., Makumbi, D., Tarekegne, A., Das, B., Zaman-Allah, M., & Cairns, J. E.  (2017). Gains in maize genetic improvement in Eastern and Southern Africa: I CIMMYT hybrid breeding pipeline. Crop Sci.

[CR77] Meng T, Ge J, Zhang X, Chen X, Zhou G, Wei H (2021). Improvements in plant morphology facilitating progressive yield increases of japonica Inbred Rice since the 1980s in East China. Agriculture.

[CR78] Meng T, Zhang X, Ge J, Chen X, Zhu G, Chen Y, Zhou G, Wei H, Dai Q (2022). Improvements in grain yield and nutrient utilization efficiency of japonica inbred rice released since the 1980s in eastern China. Field Crop Res.

[CR79] Meuwissen TH, Hayes BJ, Goddard ME (2001). Prediction of total genetic value using genome-wide dense marker maps. Genetics.

[CR80] Miura K, Ashikari M, Matsuoka M (2011). The role of QTLs in the breeding of high-yielding rice. Trends Plant Sci.

[CR81] Morais Júnior OP, Breseghello F, Duarte JB, Morais OP, Rangel PHN, Coelho ASG (2017). Effectiveness of recurrent selection in irrigated rice breeding. Crop Sci.

[CR82] Muleta KT, Pressoir G, Morris GP (2019). Optimizing genomic selection for a sorghum breeding program in Haiti: a simulation study. G3 Genes Genomes Genet.

[CR83] Müller D, Schopp P, Melchinger AE (2017). Persistency of prediction accuracy and genetic gain in synthetic populations under recurrent genomic selection. G3.

[CR84] Muralidharan K, Prasad GSV, Rao CS (1996). Breeding for rice improvement: Where do we stand?. Curr Sci.

[CR85] Muralidharan K, Prasad GSV, Rao CS, Siddiq EA (2019). Genetic gain for yield in rice breeding and rice production in India to meet with the demand from increased human population. Curr Sci.

[CR86] Muralidharan K, Prasad GSV, Rao CS, Sridhar R, Siddiq EA (2022). Grain yield performance of hybrid rice in relation to inbred cultivars in long-term multi-environment tests in India. Crop Sci.

[CR87] Muralidharan K, Prasad GS, Rao C (2002) Yield performance of rice genotypes in international multi-environment trials during 1976–97. Undefined. https://www.semanticscholar.org/paper/Yield-performance-of-rice-genotypes-in-trials-Muralidharan-Prasad/baddf1cd13d1caee866c634359952c7001072b30

[CR88] Nguyen NV, Ferrero A (2006). Meeting the challenges of global rice production. Paddy Water Environ.

[CR89] Peleman JD, van der Voort JR (2003). Breeding by design. Trends Plant Sci.

[CR90] Peng S, Khushg G (2003). Four decades of breeding for varietal improvement of irrigated lowland rice in the International Rice Research Institute. Plant Prod Sci.

[CR91] Peng S, Laza RC, Visperas RM, Sanico AL, Cassman KG, Khush GS (2000). Grain Yield of rice cultivars and lines developed in the Philippines since 1966. Crop Sci.

[CR92] Peng S, Khush GS, Cassman KG (1994) Evolution of the new plant ideotype for increased yield potential. In: Cassman KG (Ed), Breaking the yield barrier: proceedings of a workshop on rice yield potential in favorable environments, pp 5–20. IRRI

[CR93] Peng S, Laza RC, Visperas RM, Khush GS, Virk P, Zhu D (2004) Rice: progress in breaking the yield ceiling 11

[CR94] Peng S, Khush GS, Virk P, Tang Q, Zou Y (2008) Progress in ideotype breeding to increase rice yield potential. Field Crops Res 7

[CR95] Pereira de Castro A, Breseghello F, Furtini IV, Utumi MM, Pereira JA, Cao T-V, Bartholomé J (2023). Population improvement via recurrent selection drives genetic gain in upland rice breeding. Heredity.

[CR96] Phocas F (2011) L’optimisation des programmes de sélection. INRAE Prod Anim 24(4) :341–356. 10.20870/productions-animales.2011.24.4.3266

[CR97] Piepho H-P, Laidig F, Drobek T, Meyer U (2014). Dissecting genetic and non-genetic sources of long-term yield trend in German official variety trials. Theor Appl Genet.

[CR98] Pinson SRM, Jia Y, Gibbons J (2012). Response to early generation selection for resistance to rice kernel fissuring. Crop Sci.

[CR99] Platten JD, Fritsche-Neto R (2022) Optimizing QTL introgression via stochastic simulations: an example of the IRRI rice breeding program. 10.21203/rs.3.rs-1780978/v1

[CR100] Pook T, Schlather M, Simianer H (2020). MoBPS—modular breeding program simulator. G3 Genes Genomes Genet.

[CR101] Rahman NMd, F., Malik, W. A., Kabir, Md. S., Baten, Md. A., Hossain, Md. I., Paul, D. N. R., Ahmed, R., Biswas, P. S., Rahman, Md. C., Rahman, Md. S., Iftekharuddaula, K. Md., Hadasch, S., Schmidt, P., Islam, Md. R., Rahman, Md. A., Atlin, G. N., & Piepho, H.-P.  (2023). 50 years of rice breeding in Bangladesh: genetic yield trends. Theor Appl Genet.

[CR102] Rangel PHN, Pereira JA, Morais OPD, Guimarães EP, Yokokura T (2000). Genetic gains for grain yield by irrigated rice breeding program in the mid-north region of Brazil. Pesq Agrop Brasileira.

[CR103] Ray DK, Ramankutty N, Mueller ND, West PC, Foley JA (2012). Recent patterns of crop yield growth and stagnation. Nat Commun.

[CR104] Ray DK, Mueller ND, West PC, Foley JA (2013). Yield trends are insufficient to double global crop production by 2050. PLoS ONE.

[CR105] Rutkoski JE (2019). Estimation of realized rates of genetic gain and indicators for breeding program assessment. Crop Sci.

[CR106] Rutkoski JE (2019a) Chapter four—a practical guide to genetic gain. In: Sparks DL (Ed), Advances in agronomy, Vol 157, pp 217–249. Academic Press. 10.1016/bs.agron.2019.05.001

[CR107] Sakamoto T, Matsuoka M (2008). Identifying and exploiting grain yield genes in rice. Curr Opin Plant Biol.

[CR108] Samonte SO, Andaya V, Jodari F, Andaya C, Sanchez P, Mckenzie K (2016) Yield increase rate of calrose cultivars developed by the rice experiment station from 1976 to 2015. 10.13140/RG.2.2.13182.46407

[CR109] Schuster W (1997). How much does plant breeding contribute to yield lmprovement of crops?. Pflanzenbauwissenschaften.

[CR110] Siddiq EA, Vemireddy LR (2021) Advances in genetics and breeding of rice: an overview. In: Ali J, Wani SH (Eds) Rice improvement: physiological, molecular breeding and genetic perspectives, pp 1–29. Springer International Publishing. 10.1007/978-3-030-66530-2_1

[CR111] Soares AA, Santos PG, de Morais OP, Soares PC, de Reis M, S., & Souza, M. A. de.  (1999). Genetic progress obtained by upland rice breeding in twenty one years of research in the state of Minas Gerais, Brazil. Pesq Agrop Brasileira.

[CR112] Soares PC, Melo PGS, Melo LC, Soares AA (2005) Genetic gain in an improvement program of irrigated rice in Minas Gerais. Undefined. https://www.semanticscholar.org/paper/Accuracy-and-genetic-progress-of-agronomic-traits-Gabriel-Roberto/5df86b5973ef50b52515cbb19c8582e1439f351f

[CR113] Souza MA, Morais OPD, Heran R, Cargnin A, Badaró PAJ (2007) Genetic progress of upland rice between 1950 and 2001. 10.1590/S0100-204X2007000300010

[CR114] Streck EA, de Magalhaes AM, Aguiar GA, Henrique Facchinello PK, Perin L, Reis Fagundes PR, de Oliveira AC (2018). Genetic progress of grain quality of flooded-irrigated rice cultivars in the state of Rio Grande do Sul, Brazil. Pesq Agrop Brasileira.

[CR115] Streck EA, de Magalhaes AM, Aguiar GA, Henrique Facchinello PK, Reis Fagundes PR, Franco DF, Nardino M, de Oliveira AC (2018). Genetic progress in 45 years of irrigated rice breeding in Southern Brazil. Crop Sci.

[CR116] Sun X, Peng T, Mumm RH (2011). The role and basics of computer simulation in support of critical decisions in plant breeding. Mol Breed.

[CR117] Tabien RE, Samonte SOPB, McClung AM (2008). Forty-eight years of rice improvement in Texas since the release of cultivar Bluebonnet in 1944. Crop Sci.

[CR118] Tanaka J, Hayashi T, Iwata H (2016). A practical, rapid generation-advancement system for rice breeding using simplified biotron breeding system. Breed Sci.

[CR119] van Oort PAJ, Zwart SJ (2018). Impacts of climate change on rice production in Africa and causes of simulated yield changes. Glob Change Biol.

[CR120] Venkatanagappa S, Collard BCY, Gulles A, Rafiq Islam M, Lopena V, Pamplona A (2021) Assessment of genetic gain trends for yield in IRRI Rice varieties in the Philippines Using “era” trial studies and implications for future rice breeding. 10.21203/rs.3.rs-1046247/v1

[CR121] Vergara BS, Tanaka A, Lilis R, Puranabhavung S (1966). Relationship between growth duration and grain yield of rice plants. Soil Sci Plant Nutr.

[CR122] Wang W, Mauleon R, Hu Z, Chebotarov D, Tai S, Wu Z, Li M, Zheng T, Fuentes RR, Zhang F, Mansueto L, Copetti D, Sanciangco M, Palis KC, Xu J, Sun C, Fu B, Zhang H, Gao Y, Leung H (2018). Genomic variation in 3010 diverse accessions of Asian cultivated rice. Nature.

[CR123] Witcombe JR, Gyawali S, Subedi M, Virk DS, Joshi KD (2013). Plant breeding can be made more efficient by having fewer, better crosses. BMC Plant Biol.

[CR124] Xiao YG, Qian ZG, Wu K, Liu JJ, Xia XC, Ji WQ, He ZH (2012). Genetic gains in grain yield and physiological traits of winter wheat in Shandong Province, China, from 1969 to 2006. Crop Sci.

[CR125] Xie F, Zhang J (2018). Shanyou 63: an elite mega rice hybrid in China. Rice.

[CR126] Xing Y, Zhang Q (2010). Genetic and molecular bases of rice yield. Annu Rev Plant Biol.

[CR127] Xu L, Yuan S, Man J (2020). Changes in rice yield and yield stability in China during the past six decades. J Sci Food Agric.

[CR128] Xu Y, Chu C, Yao S (2021). The impact of high-temperature stress on rice: Challenges and solutions. Crop J.

[CR129] Yadav R, Gupta S, Gaikwad KB, Bainsla NK, Kumar M, Babu P, Ansari R, Dhar N, Dharmateja P, Prasad R (2021). Genetic gain in yield and associated changes in agronomic traits in wheat cultivars developed between 1900 and 2016 for irrigated ecosystems of northwestern plain zone of India. Front Plant Sci.

[CR130] Zeleke BZ, Dejene T, Worede F (2021). Genetic gain in yield and yield attributing traits of rice under upland ecosystem of Fogera, Northwest Ethiopia. Black Sea J Agric.

[CR131] Zhou X-Q, Chen D-G, Guo J, Chen P-L, Li L-J, Chen K, Chen Y-D, Liu C-G, Zhang Z-M (2021). Genetic improvement of grain quality traits in indica inbred rice cultivars developed in South China during 1956–2020. Euphytica.

[CR132] Zhu G, Peng S, Huang J, Cui K, Nie L, Wang F (2016). Genetic improvements in rice yield and concomitant increases in radiation- and nitrogen-use efficiency in middle reaches of Yangtze River. Sci Rep.

